# Supplementary material for: Factors associated with perinatal mortality in sub-Saharan Africa: A multilevel analysis
Source: PLoS One. 2024 Nov 21;19(11):e0314096. doi: 10.1371/journal.pone.0314096 (PMC11581209; doi:10.1371/journal.pone.0314096)
Supplement: Attachment — Submitted filename: PONE-D-24-24034_R1-AF.pdf [file pone.0314096.s003.pdf]

PLOS ONE

Factors associated with perinatal mortality in Sub-Saharan Africa: A multilevel analysis

--Manuscript Draft--

**Manuscript Number:**	PONE-D-24-24034R1
**Article Type:**	Research Article
**Full Title:**	Factors associated with perinatal mortality in Sub-Saharan Africa: A multilevel analysis
**Short Title:**	Factors associated with perinatal mortality in Sub-Saharan Africa: A multilevel analysis
**Corresponding Author:**	Meklit Melaku Bezie<br>University of Gondar<br>Gondar, ETHIOPIA
**Keywords:**	Demographic and health survey; Perinatal mortality; Multilevel Analysis; sub-Saharan Africa
**Abstract:**	Background: Perinatal mortality is a major global public health concern, especially in sub-Saharan Africa (SSA). Despite perinatal mortality being a major public health concern in SSA, there are very limited studies on the incidence and factors associated with perinatal mortality. Therefore, we aimed to investigate the factors associated with perinatal mortality in SSA.<br>Methods: A secondary data analysis was conducted based on the Demographic and Health Survey (DHS) data of 27 SSA countries. About 314,099 births in the preceding five years of the surveys were considered for the analysis. A multilevel binary logistic regression model was fitted to identify factors associated with perinatal mortality. Deviance (-2Log-Likelihood Ratio (LLR)) was used for model comparison. The Adjusted Odds Ratio (AOR) with the 5% Confidence Interval (CI) of the best-fitted model was used to verify the significant association between factors and perinatal mortality.<br>Results: The perinatal mortality rate in sub-Saharan Africa (SSA) was 37.31 per 1,000 births (95% CI: 36.65, 37.98). In the final best-fit model, factors significantly associated with higher perinatal mortality included media exposure (AOR: 1.12, 95% CI: 1.08, 1.17), maternal age ≥ 35 years (AOR: 1.13, 95% CI: 1.06, 1.21), health facility delivery (AOR: 1.10, 95% CI: 1.06, 1.15), having 2-4 births (AOR: 1.35, 95% CI: 1.25, 1.47), five or more births (AOR: 1.69, 95% CI: 1.53, 1.86), residence in West Africa (AOR: 1.30, 95% CI: 1.24, 1.36) or Central Africa (AOR: 1.05, 95% CI: 1.00, 1.11), rural residency (AOR: 1.08, 95% CI: 1.02, 1.13), and difficulty accessing a health facility (AOR: 1.06, 95% CI: 1.02, 1.10). In contrast, factors significantly associated with lower perinatal mortality were a preceding birth interval of 2-4 years (AOR: 0.70, 95% CI: 0.67, 0.74) or five or more years (AOR: 0.91, 95% CI: 0.84, 0.97), Antenatal Care (ANC) visit (AOR: 0.66, 95% CI: 0.63, 0.69), higher education levels (AOR: 0.82, 95% CI: 0.73, 0.93), middle household wealth (AOR: 0.93, 95% CI: 0.88, 0.98), and richer household wealth (AOR: 0.93, 95% CI: 0.87, 0.99)<br>Conclusion: Perinatal mortality was a major public health problem in SSA. Maternal socio-demographic, obstetrical, and healthcare-related factors are significantly associated with perinatal mortality. The findings of this study highlighted the need for holistic healthcare interventions targeting enhancing maternal healthcare services to reduce the incidence of perinatal mortality.
**Order of Authors:**	Meklit Melaku Bezie
	Hiwot Altaye Asebe
	Angwach Abrham Asnake
	Bezawit Melak Fente
	Yohannes Mekuria Negussie
	Zufan Alamrie Asmare
	Mamaru Melkam
	Beminate Lemma Seifu

Powered by Editorial Manager® and ProduXion Manager® from Aries Systems Corporation

Opposed Reviewers:	
Response to Reviewers:	Author response<br>PLOS ONE Journal<br>Manuscript title: Factors associated with perinatal mortality in Sub-Saharan Africa: A multilevel analysis<br>Manuscript ID: PONE-D-24-24034<br>Dear editor/reviewer.<br>Dear all,<br>We would like to thank you for the constructive, building, and improvable comments on this manuscript that would improve the content of the manuscript. We considered each comment and clarification question of editors and reviewers on the manuscript thoroughly. Our point-by-point responses for each comment and question are described in detail on the following pages. Further, the details of changes were shown by track changes in the supplementary document attached.<br><br>Reviewer comments<br>1.Abstract<br>These variables will have to be put in special case, e.g., Media Exposure, or with inverted commas, e.g., "Media Exposure" (as it was in the report), so that it is clear to the reader...<br>Authors' response: Thank you reviewer for the comments. We have accepted all the comments and revised them according to your suggestions. (See the Revised manuscript)<br>2.Background<br>-It would be good to put some figures here, so the reader can focus on differences between regions.<br>Authors' response: Thank you reviewer for the suggestions. We have added the figures for the perinatal mortality rate. "Annually, nearly 7 million perinatal deaths occur globally, including 3 to 4 million stillbirths and 3 million early neonatal deaths, with approximately 99% of these deaths taking place in low- and middle-income regions, predominantly in sub-Saharan Africa". (See the revised manuscript)<br>-By which institution has this been advocated...WHO?...Important to instruct the reader...<br>Authors' response: Thank you reviewer for the comment. We have included which institution advocated the ENAP. "Several interventions to lower the Perinatal Mortality Rate (PMR) have been implemented globally, aiming to reduce stillbirths and neonatal deaths to 12 per 1,000 births by 2030 through the adoption of the Every Newborn Action Plan (ENAP) [12, 26, 27]. Launched in 2014 by the World Health Organization (WHO) and the United Nations Children's Fund (UNICEF), ENAP seeks to prevent newborn deaths and stillbirths while improving maternal and child health worldwide. However, PMR in sub-Saharan Africa remains alarmingly high compared to developed nations". (See the Revised manuscript).<br>3.Methods<br>-The sentence is unclear. Here it seems to mean that the birth record dataset was also used to extract data on "independent variables". Needs rephrasing.<br>Authors' response: Thank you reviewer for the comments. We used the Births Record (BR) dataset. As you know there are several DHS datasets including the women, men, kids, births, couples, and household datasets, and the type of dataset to be used depends on the objective of the research. For the child and birth outcomes, we can use either the births record or kids record data. As per the DHS recode manual and guide to statistics, we used the BR dataset to extract the outcome and independent variables. "Data on perinatal mortality and independent variables were extracted from the Births Record (BR) dataset.".<br>-Again, variables should be written differently from the sentence words, e.g., by capitalizing them, e.g., No, Yes, or better, by using inverted commas (e.g., "Yes", "No").<br>Authors' response: Thank you reviewer for the comment. We have accepted the comment and made the revision. (See the Revised manuscript)<br>-"and deviance"? Is this an incomplete sentence? Please re-word/improve.<br>Authors' response: Thank you for the comment. We have revised it. We used the deviance (-2LLR) for model comparison because the models were nested models where the lower the deviance value the better the model fits the data. A model with the lowest deviance value was chosen as the best-fitted model. We have added some details about deviance in the methods. "Four models were fitted, and model

	comparison was conducted using deviance (-2 Log-likelihood Ratio (-2LLR)), with the lowest value indicating the best-fitting model for the data. Deviance, expressed as the -2 Log-likelihood Ratio (-2LLR), is a statistical metric used to compare nested models in logistic regression and other likelihood-based approaches [31]. For nested models, it assesses whether the more complex model provides a better fit to the data than a simpler model by determining if the additional parameters in the complex model significantly enhance its performance. When it approaches zero, it indicates that the model fits the data exceptionally well". (See the Revised manuscript)<br>-De-identified DHS data, with what?<br>Author's response: Thank you for the comment. We have revised the ethical consideration statement. In DHS, data was de-identified using data anonymization and removal of personal identifiers to ensure the participant's privacy and confidentiality. "For this study, we have received an authorization letter from the Measure DHS program for using the data. DHS provides publicly available de-identified data, so ethical approval is not needed. DHS employs several approaches to de-identify the data to ensure the privacy and confidentiality of respondents, such as data anonymization and the removal of personal identifiers". (See the Revised manuscript)<br>4.Results<br>1.For the benefit of the reader, develop/explain this more, e.g., a way to generalize the residual sums of squares, to show the "goodness of fit"...etc.<br>Author's response: Thank you for raising your concern. For our study, we used LLR and deviance for model comparison. As you may know, deviance is -2 times the log-likelihood ratio of the model. Given our outcome variable is binary /categorical, the model fitness/adequacy is ideally assessed using LLR because the model is based on the maximum likelihood estimation, unlike the linear regression where least square regression is used. So, the residual sum of squares is the key metric for assessing the model fitness of linear regression models where the residuals are the difference between the predicted and observed values. Therefore, the residual sum of squares can not be used for the logistic regression model. So, for our study, we used the model fitness using the LR test and deviance. For further reading see https://stats.stackexchange.com/questions/237702/comparing-models-using-the-deviance-and-log-likelihood-ratio-tests.<br><br>General comments<br>We have accepted all the editorial comments and revised them accordingly. (See the revised manuscript)
**Additional Information:**	
**Question**	**Response**
**Financial Disclosure**<br><br>Enter a financial disclosure statement that describes the sources of funding for the work included in this submission. Review the submission guidelines for detailed requirements. View published research articles from *PLOS ONE* for specific examples.<br><br>This statement is required for submission and **will appear in the published article** if the submission is accepted. Please make sure it is accurate.	The author(s) received no specific funding for this work.

Powered by Editorial Manager® and ProduXion Manager® from Aries Systems Corporation

**Funded studies**<br>Enter a statement with the following details:<br>• Initials of the authors who received each award<br>• Grant numbers awarded to each author<br>• The full name of each funder<br>• URL of each funder website<br>• Did the sponsors or funders play any role in the study design, data collection and analysis, decision to publish, or preparation of the manuscript?<br><br>Did you receive funding for this work?	
**Competing Interests**<br><br>Use the instructions below to enter a competing interest statement for this submission. On behalf of all authors, disclose any competing interests that could be perceived to bias this work—acknowledging all financial support and any other relevant financial or non-financial competing interests.<br><br>This statement is required for submission and **will appear in the published article** if the submission is accepted. Please make sure it is accurate and that any funding sources listed in your Funding Information later in the submission form are also declared in your Financial Disclosure statement.<br><br>View published research articles from *PLOS ONE* for specific examples.	The authors have declared that no competing interests exist.

Powered by Editorial Manager® and ProduXion Manager® from Aries Systems Corporation

**NO authors have competing interests**<br><br>Enter: *The authors have declared that no competing interests exist.*<br><br>**Authors with competing interests**<br><br>Enter competing interest details beginning with this statement:<br><br>*I have read the journal's policy and the authors of this manuscript have the following competing interests: [insert competing interests here]*<br><br>* typeset	
**Ethics Statement**<br><br>Enter an ethics statement for this submission. This statement is required if the study involved:<br><br>• Human participants<br>• Human specimens or tissue<br>• Vertebrate animals or cephalopods<br>• Vertebrate embryos or tissues<br>• Field research<br><br>Write "N/A" if the submission does not require an ethics statement.<br><br>General guidance is provided below. Consult the submission guidelines for detailed instructions. **Make sure that all information entered here is included in the Methods section of the manuscript.**	N/A

**Format for specific study types**<br><br>**Human Subject Research (involving human participants and/or tissue)**<br>• Give the name of the institutional review board or ethics committee that approved the study<br>• Include the approval number and/or a statement indicating approval of this research<br>• Indicate the form of consent obtained (written/oral) or the reason that consent was not obtained (e.g. the data were analyzed anonymously)<br><br>**Animal Research (involving vertebrate animals, embryos or tissues)**<br>• Provide the name of the Institutional Animal Care and Use Committee (IACUC) or other relevant ethics board that reviewed the study protocol, and indicate whether they approved this research or granted a formal waiver of ethical approval<br>• Include an approval number if one was obtained<br>• If the study involved *non-human primates*, add *additional details* about animal welfare and steps taken to ameliorate suffering<br>• If anesthesia, euthanasia, or any kind of animal sacrifice is part of the study, include briefly which substances and/or methods were applied<br><br>**Field Research**<br><br>Include the following details if this study involves the collection of plant, animal, or other materials from a natural setting:<br>• Field permit number<br>• Name of the institution or relevant body that granted permission	
**Data Availability**<br><br>Authors are required to make all data underlying the findings described fully available, without restriction, and from the time of publication. PLOS allows rare exceptions to address legal and ethical concerns. See the PLOS Data Policy and FAQ for detailed information.	Yes - all data are fully available without restriction

A Data Availability Statement describing where the data can be found is required at submission. Your answers to this question constitute the Data Availability Statement and **will be published in the article**, if accepted.<br><br>**Important:** Stating 'data available on request from the author' is not sufficient. If your data are only available upon request, select 'No' for the first question and explain your exceptional situation in the text box.<br><br>Do the authors confirm that all data underlying the findings described in their manuscript are fully available without restriction?	
**Describe where the data may be found in full sentences. If you are copying our sample text, replace any instances of XXX with the appropriate details.**<br><br>• If the data are **held or will be held in a public repository**, include URLs, accession numbers or DOIs. If this information will only be available after acceptance, indicate this by ticking the box below. For example: *All XXX files are available from the XXX database (accession number(s) XXX, XXX.).*<br>• If the data are all contained **within the manuscript and/or Supporting Information files**, enter the following: *All relevant data are within the manuscript and its Supporting Information files.*<br>• If neither of these applies but you are able to provide **details of access elsewhere**, with or without limitations, please do so. For example:<br><br>*Data cannot be shared publicly because of [XXX]. Data are available from the XXX Institutional Data Access / Ethics Committee (contact via XXX) for researchers who meet the criteria for access to confidential data.*<br><br>*The data underlying the results presented in the study are available from (include the name of the third party*	Data for this study were sourced from Demographic and Health surveys (DHS) and available here: https://dhsprogram.com/data/dataset_admin/login_main.cfm.

and contact information or URL).<br>• This text is appropriate if the data are owned by a third party and authors do not have permission to share the data.<br><br>* typeset	
Additional data availability information:	

Powered by Editorial Manager® and ProduXion Manager® from Aries Systems Corporation

To: **Editors in PLOS ONE Journal** Date: June 2024

Subject **<u>Submitting Manuscript</u>**

We are pleased to submit an original research article entitled "***Factors associated with perinatal mortality in Sub-Saharan Africa: A multilevel analysis***". Despite the perinatal mortality continues as a major public health problem in SSA, there are limited published studies on the magnitude and factors associated with perinatal mortality in SSA. Therefore, we aimed to investigate the magnitude and factors associated with perinatal mortality in SSA based on Demographic and Health Survey (DHS) data from 27 SSA countries. This study will guide program planners and decision-makers to design evidence-based interventions to reduce the incidence of perinatal mortality in SSA.

We consider PLOS ONE journal for publication because it is peer reviewed journal with high impact factor and fastest rate of processing. This manuscript is our original work, not published before and is not under consideration for publication any Journal elsewhere. We have no conflicts of interest to disclose.

Corresponding author

Meklit Melaku Bezie

Email: *mesiyemaki@gmail.com*

Manuscript

Click here to access/download;Manuscript;Revised manuscript.docx

Factors associated with perinatal mortality in Sub-Saharan Africa: A multilevel analysis

Meklit Melaku Bezie[1], Hiwot Altaye Asebe[2], Angwach Abrham Asnake[3], Bezawit Melak Fente[4], Yohannes Mekuria Negussie[5], Zufan Alamrie Asmare[6], Mamaru Melkam[7], Beminate Lemma Seifu[8]

[1]Department of Public Health, Institute of Public Health, College of Medicine and Health Sciences and Comprehensive Specialized Hospital, University of Gondar, Gondar, Ethiopia

[2]Department of Public Health, College of Medicine and Health Sciences, Samara University, Samara, Ethiopia

[3]Department of Epidemiology and Biostatistics, School of Public Health, College of Medicine and Health Sciences, Wolaita Sodo University, Wolaita Sodo, Ethiopia

[4]Department of General Midwifery, School of Midwifery, College of Medicine & Health Sciences, University of Gondar, Gondar, Ethiopia

[5]Department of Medicine, Adama General Hospital and Medical College, Adama, Ethiopia

[6]Department of Ophthalmology, School of Medicine and Health Science, Debre Tabor University, Debre Tabor, Ethiopia.

[7]University of Gondar College of Medicine and Health Science, Department of Psychiatry, Gondar, Ethiopia

[8]Department of Public Health, College of Medicine and Health Sciences, Samara University, 21 Samara, Ethiopia

Corresponding Author: Meklit Melaku Bezie

Email: mesiyemaki@gmail.com

Abstract

Background: Perinatal mortality is a major global public health concern, especially in sub-Saharan Africa (SSA). Despite perinatal mortality being a major public health concern in SSA, there are very limited studies on the incidence and factors associated with perinatal mortality. Therefore, we aimed to investigate the factors associated with perinatal mortality in SSA.

Methods: A secondary data analysis was conducted based on the Demographic and Health Survey (DHS) data of 27 SSA countries. About 314,099 births in the preceding five years of the surveys were considered for the analysis. A multilevel binary logistic regression model was fitted to identify factors associated with perinatal mortality. Deviance (-2Log-Likelihood Ratio (LLR)) was used for model comparison. The Adjusted Odds Ratio (AOR) with the 5% Confidence Interval (CI) of the best-fitted model was used to verify the significant association between factors and perinatal mortality.

Results: The perinatal mortality rate in sub-Saharan Africa (SSA) was 37.31 per 1,000 births (95% CI: 36.65, 37.98). In the final best-fit model, factors significantly associated with higher perinatal mortality included media exposure (AOR: 1.12, 95% CI: 1.08, 1.17), maternal age ≥ 35 years (AOR: 1.13, 95% CI: 1.06, 1.21), health facility delivery (AOR: 1.10, 95% CI: 1.06, 1.15), having 2-4 births (AOR: 1.35, 95% CI: 1.25, 1.47), five or more births (AOR: 1.69, 95% CI: 1.53, 1.86), residence in West Africa (AOR: 1.30, 95% CI: 1.24, 1.36) or Central Africa (AOR: 1.05, 95% CI: 1.00, 1.11), rural residency (AOR: 1.08, 95% CI: 1.02, 1.13), and difficulty accessing a health facility (AOR: 1.06, 95% CI: 1.02, 1.10). In contrast, factors significantly associated with lower perinatal mortality were a preceding birth interval of 2-4 years (AOR: 0.70, 95% CI: 0.67, 0.74) or five or more years (AOR: 0.91, 95% CI: 0.84, 0.97), Antenatal Care (ANC) visit (AOR:

0.66, 95% CI: 0.63, 0.69), higher education levels (AOR: 0.82, 95% CI: 0.73, 0.93), middle household wealth (AOR: 0.93, 95% CI: 0.88, 0.98), and richer household wealth (AOR: 0.93, 95% CI: 0.87, 0.99)

Conclusion: Perinatal mortality was a major public health problem in SSA. Maternal socio-demographic, obstetrical, and healthcare-related factors are significantly associated with perinatal mortality. The findings of this study highlighted the need for holistic healthcare interventions targeting enhancing maternal healthcare services to reduce the incidence of perinatal mortality.

Keywords: Demographic and Health Survey, Perinatal mortality, Multilevel analysis, sub-Saharan Africa

Background

Perinatal mortality is defined as the death of a fetus after 28 weeks of gestation (stillbirth) plus the death of a neonate within 7 days of life [1]. Annually, over 6.9 million perinatal deaths occur globally [2-5], and almost all cases occur in Low-income and Middle-income Countries (LMICs) [5]. Despite the substantial reduction in global perinatal mortality [6], LMICs such as sub-Saharan African (SSA) countries continue sharing the huge burden of global perinatal deaths [7]. Annually, nearly 7 million perinatal deaths occur globally, including 3 to 4 million stillbirths and 3 million early neonatal deaths, with approximately 99% of these deaths taking place in low- and middle-income regions, predominantly in sub-Saharan Africa [8]

LMICs are still seemingly walking a patchy road towards achieving the Sustainable Development Goal (SDG), which is to end preventable deaths of under-five children in 2023 [9]. Of the global 6.9 million perinatal deaths, the vast majority (98%) of these deaths were reported to have occurred in SSA [10]. Over three-quarters of perinatal mortality is due to preventable causes [11, 12]. The perinatal mortality rate indicates the accessibility and quality of healthcare for the mother and baby [13]. For millions of families in low- and middle-income nations, perinatal mortality continues to be one of the most devastating pregnancy outcomes [14]. Currently, available evidence shows that perinatal mortality reflects the quality of maternal healthcare services during pregnancy, delivery, and postnatal care [15, 16]. It results from the complex interaction of individual-level factors linked to maternal lifestyle and obstetric complications, which could be exacerbated by underlying community-level factors such as poor access to quality maternal and newborn health services.

Perinatal mortality is a key indicator of women's health-seeking behavior, maternal healthcare access, socioeconomic status, and the standard of obstetric care [17]. Beyond losing the child, perinatal mortality causes emotional and psychological stress for the mother. Mother may also experience social stigma or strained relationships with their partners [18]. In addition, it has a significant financial cost, worsens the health of the mother, perpetuates the cycle of poverty, and impedes social and economic development, especially in Sub-Saharan Africa (SSA), where child mortality is eleven times higher than in developed nations [19].

Previous studies published on determinants of perinatal mortality revealed that maternal education [20], parity [21], residence [22], preceding birth interval [23], place of delivery [24], number of Antenatal Care (ANC) [22], media exposure [25] and wealth status were significantly associated with perinatal mortality. Several interventions to lower the Perinatal Mortality Rate (PMR) have been implemented globally, aiming to reduce stillbirths and neonatal deaths to 12 per 1,000 births by 2030 through the adoption of the Every Newborn Action Plan (ENAP) [12, 26, 27]. Launched in 2014 by the World Health Organization (WHO) and the United Nations Children's Fund (UNICEF), ENAP seeks to prevent newborn deaths and stillbirths while improving maternal and child health worldwide. However, PMR in sub-Saharan Africa remains alarmingly high compared to developed nations [28].

Despite perinatal mortality continuing to be a major public health problem in SSA, there are limited published studies on the magnitude and factors associated with perinatal mortality in SSA. Therefore, we aimed to investigate the magnitude and factors associated with perinatal mortality in SSA based on Demographic and Health Survey (DHS) data from 27 SSA countries. This study will guide program planners and decision-makers in designing evidence-based interventions to reduce perinatal mortality in SSA.

Methods

Study design and sample

We analyzed DHS data from 27 sub-Saharan African countries. The DHS data is collected every five years to update basic health and health-related indicators of the country. It is assumed to be nationally representative, where a multi-stage stratified sampling is employed to recruit representative samples. The Enumeration Area (EA) served as the primary sampling unit, while households were the secondary sampling unit. Data on perinatal mortality and independent variables were extracted from the Births Record (BR) dataset. Key variables from 27 sub-Saharan African (SSA) countries were collected and combined for the final analysis. The dataset included a total of 314,099 births from women of reproductive age within the five years preceding the survey.

Dependent variable

The dependent variable for this study was perinatal mortality. Perinatal mortality was a composite of stillbirth and early neonatal death (deaths occurred before 7 days of birth) [29, 30]. The outcome variable (Perinatal Mortality) was binary and recoded as "Yes" and "No".

Covariates

We used both individual- and community-level variables for our study. Maternal Age (grouped as 15 – 24, 25 – 34, and 35 -49 years), Media Exposure (grouped as No and Yes), Household Wealth Status (grouped as Poorest, Poorer, Middle, Richer, and Richest), Preceding Birth Interval (grouped as < 2 years, 2 – 4 years, ≥5 years and Primiparous), Place of Delivery (grouped as Home and Health Facility), Parity (grouped as One, Two – Four and Five births or above), ANC

visits (grouped as No and Yes), Maternal Educational Status (grouped as No, Primary, Secondary and Higher Education), and Maternal Working Status (grouped as No and Yes) were the individual-level variables. Whereas, Residence (grouped as Urban and Rural), Distance from a Health Facility (grouped as a big problem and not a big problem), and SSA region (grouped as central, eastern, western, and southern regions) were considered as community-level variables.

Data management and analysis

To ensure the study results were representative, we used DHS sample weights to account for unequal probability sampling across strata and non-response. Descriptive characteristics including frequency and proportion were reported based on the weighted data to describe the study population. A multilevel binary logistic regression model was fitted to account for both the individual- and community-level variables. Stata version 17 was used for data management and analysis.

A multilevel binary logistic regression model was employed to account for the hierarchical structure of DHS data. The Intra-cluster Correlation Coefficient (ICC), Median Odds Ratio (MOR), and Likelihood Ratio (LR) tests were calculated to evaluate the variability of perinatal mortality across clusters. Four models were fitted, and model comparison was conducted using deviance (-2 Log-likelihood Ratio (-2LLR)), with the lowest value indicating the best-fitting model for the data. Deviance, expressed as the -2 Log-likelihood Ratio (-2LLR), is a statistical metric used to compare nested models in logistic regression and other likelihood-based approaches [31]. For nested models, it assesses whether the more complex model provides a better fit to the data than a simpler model by determining if the additional parameters in the complex model significantly enhance its performance. When it approaches zero, it indicates that the model fits the

data exceptionally well. Model I was the null model fitted without covariates, Model II was the model with individual-level variables, Model 3 was the model with community-level variables and Model IV was the model with both individual and community-level variables. Variables with a P-value ≤ 0.2 in the bivariable analysis were selected for multivariable multilevel logistic regression analysis. In the final best-fitted model, the Adjusted Odds Ratio (AOR) with 95% Confidence Interval (CI) was used to establish the statistical significance and strength of the association.

Ethical consideration

For this study, we have received an authorization letter from the Measure DHS program for using the data. DHS provides publicly available de-identified data, so ethical approval is not needed. DHS employs several approaches to de-identify the data to ensure the privacy and confidentiality of respondents, such as data anonymization and the removal of personal identifiers.

Results

Descriptive characteristics of study participants

A total weighted sample of 314,099 births in the last five years preceding the survey was included (Table 1). More than two-thirds (68%) of participants resided in rural areas. About 110,470 (35.17%) and 109,700 (35%) of the mothers didn't have formal education and primary education, respectively. Approximately half of the mothers (47.65%) were within the age range of 25-34 years. Nearly one-fourth (22.9%) and 16.8% of participants belonged to the poorest and richest households, respectively. Only 13.33% of mothers had a history of terminating a pregnancy (Table 2).

The perinatal mortality rate in SSA

The perinatal mortality rate in SSA was 37.31 per 1000 births (95% CI: 36.65, 37.98). It has varied across countries ranging from the lowest in Mozambique (10.8 per 1000 births) to the highest in Cote D'Ivoire (68.1 per 1000 births) (Figure 1).

Factors associated with perinatal mortality

Random effect analysis results

We examined whether the multilevel binary logistic regression model was significant over the single-level binary logistic regression model using the Likelihood Ratio (LR) test. The LR test was statistically significant ($p<0.05$), indicating that the multilevel binary logistic regression model was best fitted to the single-level binary logistic regression analysis. Model comparison was employed by using deviance since models are nested models. Four models (mode I: 102197.9, model II: 100932, model III: 101997.6, and model IV: 100780.1) were fitted and model IV value (100780.1) was selected as the best-fitted model to explain perinatal mortality in SSA since it had the lowest deviance value. The MOR was 1.24 showing that if a mother of a child moved from a cluster with a low risk of perinatal mortality to a cluster with a high risk, the odds of experiencing perinatal mortality increased by 1.24 times.

Fixed effect analysis results

In multivariable multilevel logistic regression analysis, individual-level variables such as maternal age, maternal education, parity, ANC, preceding birth interval, and place of delivery, and community-level variables such as residence, distance to the health facility, and sub-Saharan African region had a statistically significant association with perinatal mortality.

The odds of perinatal mortality of babies to mothers aged 35 – 49 years were 1.13 times (AOR = 1.13, 95% CI: 1.06, 1.21) higher compared to those born to mothers aged 15-24 years. The odds

of experiencing perinatal mortality among mothers who had higher education were decreased by 18% (AOR = 0.82, 95% CI: 0.73, 0.93) compared to mothers who had no formal education. The odds of perinatal mortality among mothers who belonged to middle and richer household wealth were decreased by 7% (AOR = 0.93, 95% CI: 0.88, 0.98) and 7% (AOR = 0.93, 95% CI: 0.87, 0.99) compared to those belonged to the poorest household, respectively. Mothers who had no media exposure had 1.12 times (AOR = 1.12, 95% CI: 1.08, 1.17) higher odds of perinatal mortality compared to those who had media exposure. Multiparous and grand multiparous mothers had 1.35 times (AOR = 1.35, 95% CI: 1.25, 1.47) and 1.69 times (AOR = 1.69, 95% CI: 1.53, 1.86) higher odds of perinatal mortality compared to primiparous mother, respectively.

The odds of perinatal mortality among mothers who had preceding birth interval of 2 – 4 years and 5 years or above were decreased by 30% (AOR = 0.70, 95% CI: 0.67, 0.74) and 9% (AOR = 0.91, 95% CI: 0.84, 0.97) compared to those who had birth interval of less than 2 years, respectively. Mothers who gave birth at a health facility had 1.10 times (AOR = 1.10, 95% CI: 1.06, 1.15) higher odds of perinatal mortality than those who gave birth at home. The odds of perinatal mortality among mothers who had ANC visits were decreased by 34% (AOR = 0.66, 95% CI: 0.63, 0.69). Being a rural resident increased the odds of perinatal mortality by 8 % (AOR = 1.08, 95% CI: 1.02, 1.13). Mothers in West Africa and Central Africa had 1.30 times (AOR = 1.30, 95% CI: 1.24, 1.36) and 1.05 times (AOR = 1.05, 95% CI: 1.00, 1.11) higher odds of perinatal mortality compared to mothers in East Africa, respectively. Mothers who had big problems accessing health care increased the odds of perinatal mortality by 6% (AOR = 1.06, 95% CI: 1.02, 1.10) (Table 3).

Discussion

In our study, the perinatal mortality rate in SSA was found to be 37 per 1000 births which varied across regions. It was higher than a study reported in Indonesia [32] and India [33]. This difference may be due to the extensive socio-political and economic crises that sub-Saharan African countries have been experiencing, which are caused by both man-made and natural disasters that can contribute to increased perinatal mortality [34]. The findings of our study demonstrated that perinatal mortality in SSA remains a significant public health problem. The higher perinatal mortality rate is the result of a multiplicity of factors such as limited access to ANC, lack of quality of care during delivery, higher prevalence of poverty, low level of maternal education, maternal nutritional deficiency, teenage pregnancy, high prevalence of maternal infections (HIV, malaria, syphilis…etc), preterm births, low birth weight, and home delivery [35, 36]. Additionally, in many African countries, entrenched cultural beliefs and practices affect maternal and neonatal care, such as the delayed initiation of breastfeeding, which can impact perinatal outcomes [37].

In the multilevel analysis variables such as media exposure, maternal age, parity, household wealth status, preceding birth interval, maternal education, place of delivery, ANC, distance to health facility, residence, and SSA region were significantly associated with perinatal mortality. The odds of perinatal mortality among mothers aged 35 years and above were higher compared to those aged 15-24 years. This finding is consistent with previous studies [38, 39], likely due to the strong association between advanced maternal age and an elevated risk of adverse pregnancy conditions. These conditions include pregnancy-induced hypertension, antepartum hemorrhage, cesarean delivery, preterm delivery, low birth weight, and premature rupture of membranes, all of which can increase the risk of perinatal deaths [40, 41]. Maternal education reduces the risk of perinatal mortality. This finding was in line with studies reported in Nigeria [42], Spain [43], and Pakistan

[44]. It could be due to maternal education plays a significant role in improving maternal and perinatal health outcomes [45]. Educated mothers understand health-related information, are more likely to seek perinatal care, recognize danger signs of pregnancy, and take timely interventions [46]. In addition, educated mothers are highly likely to attend prenatal care early and regularly as well as more likely to give birth in healthcare facilities. Furthermore, mothers with higher education are empowered to make their own decisions about their health and childbirth.

The odds of perinatal mortality are high among mothers with five or more children compared to babies born to mothers who have fewer than two children. This study is in line with previous findings in Australia which stated that grand multiparous mothers are at risk of perinatal [47, 48]. This may be due to multiparous mothers undergoing uterine and placental changes that increase the risk of adverse outcomes. Poor maternal nutrition can lead to low birth weight and higher stillbirth risk. Additionally, financial constraints in low-resource settings may limit access to quality health services for these mothers.

As the birth interval increases, it is significantly associated with lower perinatal deaths, which is consistent with studies in Bangladesh [49] and Ethiopia[23]. It could be due to having a longer preceding birth interval enable the mother to recover physically and psychologically, and helps their nutritional recovery which strengthens the mother's immunity system, which in turn helps to avoid maternal infection as well as that of the fetus [50]. ANC reduces the risk of perinatal mortality. This finding is consistent with the study findings in Ethiopia [51, 52], and Gambia [53]. The possible explanation might be due to ANC makes it possible to track fetal development in order to provide timely interventions and preventive measures, as well as to diagnose pregnancy complications such as gestational diabetes and preeclampsia early on [54]. It creates the opportunity to connect mothers with health professionals, helping them prepare for birth and any

emergencies [55]. Our study revealed that perinatal mortality was significantly higher in rural areas. This is consistent with the study done in India [56]. This could be due to the majority of rural mothers giving birth at home, so they are at higher risk of intrapartum and early postpartum complications [57].

Mothers who had big problems accessing healthcare had higher odds of perinatal mortality. This study is in line with findings in Ethiopia [58, 59]. The rise in missed appointments and the postponed start of maternity care could serve as justification for this [60]. Furthermore, it can be challenging to promptly address complications like hemorrhage and obstructed labor in low socioeconomic households where transportation is a major challenge [61].

According to our findings, higher socio-economic class is associated with lower perinatal mortality. This is supported by a study done in the Netherlands [62], and in western China [63]. Our study states that babies born in health facilities had an increased risk of perinatal mortality as compared to babies born at home. This study supported the study done in Kenya [64]. This may be explained by the fact that babies born in health facilities often come from mothers who are already triaged as high-risk, inherently increasing the perinatal death rate compared to mothers with uncomplicated pregnancies who deliver at home. Additionally, usually due to transportation and other related constraints mothers in SSA mothers who arrive at health facilities [65, 66], which in turn made them at increased risk of complications such as fetal distress, meconium aspiration, .etc.

Strengths and limitations of the study

The main strength of this study is the use used representative data from DHS of 27 sub-Saharan African countries, which involves a large sample size. This study also employed a multilevel

technique that considers the hierarchical nature of the survey and therefore, the results are reliable and robust. However, the data is cross-sectional, and the temporal relationship of causations cannot be established. This study might have a recall bias, for variables such as preceding birth, postnatal care, and antenatal care visits, which should be taken into account when interpreting our results.

Conclusion

Perinatal mortality remains a significant public health concern in SSA. Advanced maternal age, high parity, being rural, having no media exposure, home delivery, and a big problem accessing health care were significantly associated with higher odds of perinatal mortality, while ANC, higher maternal education, belonging to a wealthier household, optimal and long preceding birth interval were significantly associated with lower odds of perinatal mortality. The findings of this study highlighted the need to enhance maternal education, ANC use, health facility delivery, and media exposure to reduce the incidence of perinatal mortality.

Abbreviations

ANC: Antenatal Care; AOR: Adjusted Odds Ratio; CI: Confidence interval; DHS: Demographic and Health Survey; EA: Enumeration Area; PSU: Primary Sampling Unit WHO: World Health Organization: SSA: Sub-Saharan Africa

Acknowledgments

We acknowledge the MEASURE DHS program for permitting the use of the dataset.

Authors' contributions

Conceptualization: MMB, HAA, AAA, BMF, YMN, ZAA, MM and BLS

Data curation: MMB, HAA, AAA, BMF, YMN, ZAA, MM and BLS

Formal analysis: MMB, HAA, AAA, BMF, YMN, ZAA, MM and BLS

Funding acquisition: MMB, HAA, AAA, BMF, YMN, ZAA, MM and BLS

Investigation: MMB

Methodology: MMB, HAA, AAA, BMF, YMN, ZAA, MM and BLS

Project administration: MMB, HAA, AAA, BMF, YMN, ZAA, MM and BLS

Resources: MMB, HAA, AAA, BMF, YMN, ZAA, MM and BLS

Software: MMB

Supervision: MMB, HAA, AAA, BMF, YMN, ZAA, MM and BLS

Validation: MMB, HAA, AAA, BMF, YMN, ZAA, MM and BLS

Visualization: MMB, HAA, AAA, BMF, YMN, ZAA, MM and BLS

Writing – original draft: MMB

Writing – review & editing: MMB, HAA, AAA, BMF, YMN, ZAA, MM and BLS. All authors critically reviewed and revised the article, and gave final approval for publication.

References

1. Organization WH: **Neonatal and perinatal mortality: country, regional and global estimates**: World Health Organization; 2006.
2. Berhan Y, Berhan A: **Perinatal mortality trends in Ethiopia**. *Ethiopian journal of health sciences* 2014, **24**:29-40.
3. Organization WH: **Every newborn: an action plan to end preventable deaths**. 2014.
4. Hug L, Mishra A, Lee S, You D, Moran A, Strong KL, Cao B: **A neglected tragedy the global burden of stillbirths: report of the UN inter-agency group for child mortality estimation, 2020**. In.: United Nations Children's Fund; 2020.
5. Hussain S: **Neonatal morbidity and mortality pattern in a tertiary care neonatal unit of a teaching hospital**. *Ann Pak Inst Med Sci* 2014, **10**(1):7-11.
6. Hug L, You D, Blencowe H, Mishra A, Wang Z, Fix MJ, Wakefield J, Moran AC, Gaigbe-Togbe V, Suzuki E: **Global, regional, and national estimates and trends in stillbirths from 2000 to 2019: a systematic assessment**. *The Lancet* 2021, **398**(10302):772-785.
7. Smith AC, Mutangiri W, Fox R, Crofts JF: **Millennium Development Goal 4: reducing perinatal and neonatal mortality in low-resource settings**. *Obstetrician & Gynaecologist* 2014, **16**(1).
8. Ogunlesi TA, Adetoro OO: **Preventing Perinatal Mortality in the Developing Countries**. *Contemporary Obstetrics and Gynecology for Developing Countries* 2021:23-34.
9. Bossman E: **The post-2015 development agenda: progress towards sustainable development goal target on maternal mortality and child mortality in limited resource settings with mHealth interventions: a systematic review in Sub-Saharan Africa and Southern Asia**. UiT Norges arktiske universitet; 2019.
10. Aminu M, Bar-Zeev S, White S, Mathai M, van Den Broek N: **Understanding cause of stillbirth: a prospective observational multi-country study from sub-Saharan Africa**. *BMC pregnancy and childbirth* 2019, **19**:1-10.
11. Bhutta ZA, Das JK, Bahl R, Lawn JE, Salam RA, Paul VK, Sankar MJ, Blencowe H, Rizvi A, Chou VB: **Can available interventions end preventable deaths in mothers, newborn babies, and stillbirths, and at what cost?** *The Lancet* 2014, **384**(9940):347-370.
12. Organization WH: **Reaching the every newborn national 2020 milestones: country progress, plans and moving forward**. 2017.
13. Santosh A, Zunjarwad G, Hamdi I, Al-Nabhani JA, Sherkawy BE, Al-Busaidi IH: **Perinatal mortality rate as a quality indicator of healthcare in Al-Dakhiliyah Region, Oman**. *Sultan Qaboos University Medical Journal* 2013, **13**(4):545.
14. Karlsen S, Say L, Souza J-P, Hogue CJ, Calles DL, Gülmezoglu AM, Raine R: **The relationship between maternal education and mortality among women giving birth in health care institutions: analysis of the cross sectional WHO Global Survey on Maternal and Perinatal Health**. *BMC public health* 2011, **11**:1-10.
15. Beksinska M, Kunene B, Mullick S: **Maternal care: antenatal, peri and postnatal: women's health**. *South African health review* 2006, **2006**(1):297-314.
16. Tunçalp Ö, Were W, MacLennan C, Oladapo O, Gülmezoglu A, Bahl R, Daelmans B, Mathai M, Say L, Kristensen F: **Quality of care for pregnant women and newborns—the WHO vision**. *Bjog* 2015, **122**(8):1045.
17. Schmiegelow C, Minja D, Oesterholt M, Pehrson C, Suhrs HE, Boström S, Lemnge M, Magistrado P, Rasch V, Lusingu J: **Factors associated with and causes of perinatal mortality in northeastern Tanzania**. *Acta obstetricia et gynecologica Scandinavica* 2012, **91**(9):1061-1068.
18. Fernández-Sola C, Camacho-Ávila M, Hernández-Padilla JM, Fernández-Medina IM, Jiménez-López FR, Hernández-Sánchez E, Conesa-Ferrer MB, Granero-Molina J: **Impact of perinatal death**

on the social and family context of the parents. *International journal of environmental research and public health* 2020, **17**(10):3421.

19. Schwartz DA: **Challenges in improvement of perinatal health in developing nations: Role of perinatal pathology**. *Archives of Pathology and Laboratory Medicine* 2013, **137**(6):742-746.
20. Tesema GA, Worku MG: **Individual-and community-level determinants of neonatal mortality in the emerging regions of Ethiopia: a multilevel mixed-effect analysis**. *BMC pregnancy and childbirth* 2021, **21**:1-11.
21. Yirgu R, Molla M, Sibley L, Gebremariam A: **Perinatal mortality magnitude, determinants and causes in west Gojam: population-based nested case-control study**. *PloS one* 2016, **11**(7):e0159390.
22. Kibria GMA, Burrowes V, Choudhury A, Sharmeen A, Ghosh S, Mahmud A, Kc A: **Determinants of early neonatal mortality in Afghanistan: an analysis of the Demographic and Health Survey 2015**. *Globalization and health* 2018, **14**:1-12.
23. Jena BH, Biks GA, Gelaye KA, Gete YK: **Magnitude and trend of perinatal mortality and its relationship with inter-pregnancy interval in Ethiopia: a systematic review and meta-analysis**. *BMC pregnancy and childbirth* 2020, **20**:1-13.
24. Nankabirwa V, Tumwine JK, Tylleskär T, Nankunda J, Sommerfelt H, Consortium PER: **Perinatal mortality in eastern Uganda: a community based prospective cohort study**. *PloS one* 2011, **6**(5):e19674.
25. Andargie G, Berhane Y, Worku A, Kebede Y: **Predictors of perinatal mortality in rural population of Northwest Ethiopia: a prospective longitudinal study**. *BMC public health* 2013, **13**:1-7.
26. Organization WH: **Every newborn action plan: progress report, May 2015**. 2015.
27. Plan ENA: **Every newborn: an action plan to end preventable deaths**. *Every Newborn Action Plan* 2014.
28. Wang H, Bhutta ZA, Coates MM, Coggeshall M, Dandona L, Diallo K, Franca EB, Fraser M, Fullman N, Gething PW: **Global, regional, national, and selected subnational levels of stillbirths, neonatal, infant, and under-5 mortality, 1980–2015: a systematic analysis for the Global Burden of Disease Study 2015**. *The Lancet* 2016, **388**(10053):1725-1774.
29. Smith GC, Fretts RC: **Stillbirth**. *The Lancet* 2007, **370**(9600):1715-1725.
30. Lehtonen L, Gimeno A, Parra-Llorca A, Vento M: **Early neonatal death: a challenge worldwide**. In: *Seminars in Fetal and Neonatal Medicine: 2017*: Elsevier; 2017: 153-160.
31. Quintero A, Lesaffre E: **Comparing hierarchical models via the marginalized deviance information criterion**. *Statistics in Medicine* 2018, **37**(16):2440-2454.
32. Sariunita N, Idris H: **Regional perinatal mortality differences in Indonesia: Evidence from Indonesian demographic health survey**. *Public Health in Practice* 2024, **7**:100501.
33. Misra A, Menon G, Sinha AP, Singh S, Rao MVV, Sharma S: **Risk factors for perinatal mortality in India: a systematic review of observational studies**. *International Journal of Community Medicine and Public Health* 2022, **9**(10):3849.
34. Wise PH, Darmstadt GL: **Confronting stillbirths and newborn deaths in areas of conflict and political instability: a neglected global imperative**. *Paediatrics and International Child Health* 2015, **35**(3):220-226.
35. Pervin J, Moran A, Rahman M, Razzaque A, Sibley L, Streatfield PK, Reichenbach LJ, Koblinsky M, Hruschka D, Rahman A: **Association of antenatal care with facility delivery and perinatal survival–a population-based study in Bangladesh**. *BMC pregnancy and childbirth* 2012, **12**:1-12.
36. Ntambue AM, Malonga FK, Dramaix-Wilmet M, Ngatu RN, Donnen P: **Better than nothing? maternal, newborn, and child health services and perinatal mortality, Lubumbashi, democratic republic of the Congo: a cohort study**. *BMC pregnancy and childbirth* 2016, **16**:1-12.

37. Cham M, Sundby J, Vangen S: **Maternal mortality in the rural Gambia, a qualitative study on access to emergency obstetric care**. *Reproductive health* 2005, **2**:1-8.
38. Mutz-Dehbalaie I, Scheier M, Jerabek-Klestil S, Brantner C, Windbichler GH, Leitner H, Egle D, Ramoni A, Oberaigner W: **Perinatal mortality and advanced maternal age**. *Gynecologic and obstetric investigation* 2014, **77**(1):50-57.
39. Salem Yaniv S, Levy A, Wiznitzer A, Holcberg G, Mazor M, Sheiner E: **A significant linear association exists between advanced maternal age and adverse perinatal outcome**. *Archives of gynecology and obstetrics* 2011, **283**:755-759.
40. Saccone G, Gragnano E, Ilardi B, Marrone V, Strina I, Venturella R, Berghella V, Zullo F: **Maternal and perinatal complications according to maternal age: A systematic review and meta-analysis**. *International Journal of Gynecology & Obstetrics* 2022, **159**(1):43-55.
41. Jacobsson B, Ladfors L, Milsom I: **Advanced maternal age and adverse perinatal outcome**. *Obstetrics & Gynecology* 2004, **104**(4):727-733.
42. Fawole AO, Shah A, Tongo O, Dara K, El-Ladan AM, Umezulike AC, Alu FE, Eniayewun AB, Fabanwo AO, Adewunmi AA: **Determinants of perinatal mortality in Nigeria**. *International Journal of Gynecology & Obstetrics* 2011, **114**(1):37-42.
43. Juárez S, Revuelta-Eugercios BA, Ramiro-Fariñas D, Viciana-Fernández F: **Maternal education and perinatal outcomes among Spanish women residing in southern Spain (2001–2011)**. *Maternal and child health journal* 2014, **18**:1814-1822.
44. Fikree FF, Gray RH: **Demographic survey of the level and determinants of perinatal mortality in Karachi, Pakistan**. *Paediatric and perinatal epidemiology* 1996, **10**(1):86-96.
45. Elo IT: **Utilization of maternal health-care services in Peru: the role of women's education**. *Health transition review* 1992:49-69.
46. Zaki A, Fouad S: **Assessment of knowledge and practices of pregnant women toward danger signs of pregnancy**. *Mansoura Nursing Journal* 2021, **8**(1):13-32.
47. Bai J, Wong FW, Bauman A, Mohsin M: **Parity and pregnancy outcomes**. *American journal of obstetrics and gynecology* 2002, **186**(2):274-278.
48. Seyoum E, Bekele A, Tsegaye AT, Birhanu S: **Magnitude and determinants of adverse perinatal outcomes in Tefera Hailu Memorial Hospital, Sekota Town, northern Ethiopia**. *Global pediatric health* 2021, **8**:2333794X211015524.
49. Hossain MB, Kanti Mistry S, Mohsin M, Rahaman Khan MH: **Trends and determinants of perinatal mortality in Bangladesh**. *PloS one* 2019, **14**(8):e0221503.
50. Fortney JA, Higgins JE: **The effect of birth interval on perinatal survival and birth weight**. *Public Health* 1984, **98**(2):73-83.
51. Shiferaw K, Mengiste B, Gobena T, Dheresa M: **The effect of antenatal care on perinatal outcomes in Ethiopia: A systematic review and meta-analysis**. *PloS one* 2021, **16**(1):e0245003.
52. Dessu S, Dawit Z: **Perinatal mortality and associated factors among antenatal care attended pregnant mothers at public hospitals in Gamo Zone, Southern Ethiopia**. *Frontiers in Pediatrics* 2020, **8**:586747.
53. Kumar S, Suso A, Wood E: **Effect of antenatal care on birth outcomes in The Gambia: a propensity score matching analysis**. 2024.
54. Organization WH: **WHO recommendations on antenatal care for a positive pregnancy experience: summary: highlights and key messages from the World Health Organization's 2016 global recommendations for routine antenatal care**. In.: World Health Organization; 2018.
55. Vogel JP, Habib NA, Souza JP, Gülmezoglu AM, Dowswell T, Carroli G, Baaqeel HS, Lumbiganon P, Piaggio G, Oladapo OT: **Antenatal care packages with reduced visits and perinatal mortality: a secondary analysis of the WHO Antenatal Care Trial**. *Reproductive health* 2013, **10**:1-7.

56. Tayade Surekha A, Kumar N: **Socio demographic factors affecting perinatal mortality-A study in a rural setup**. *Parity* 2012, **11**(5.97).
57. Thomas SL, Wakerman J, Humphreys JS: **Ensuring equity of access to primary health care in rural and remote Australia-what core services should be locally available?** *International journal for equity in health* 2015, **14**:1-8.
58. Kibret GD, Demant D, Hayen A: **The effect of distance to health facility on neonatal mortality in Ethiopia**. *BMC Health Services Research* 2023, **23**(1):114.
59. Målqvist M, Sohel N, Do TT, Eriksson L, Persson L-Å: **Distance decay in delivery care utilisation associated with neonatal mortality. A case referent study in northern Vietnam**. *BMC Public Health* 2010, **10**:1-9.
60. Shukla VV, Eggleston B, Ambalavanan N, McClure EM, Mwenechanya M, Chomba E, Bose C, Bauserman M, Tshefu A, Goudar SS: **Predictive modeling for perinatal mortality in resource-limited settings**. *JAMA network open* 2020, **3**(11):e2026750-e2026750.
61. Abu-Ghanem S, Sheiner E, Sherf M, Wiznitzer A, Sergienko R, Shoham-Vardi I: **Lack of prenatal care in a traditional community: trends and perinatal outcomes**. *Archives of gynecology and obstetrics* 2012, **285**:1237-1242.
62. Vidiella-Martin J, Been JV, Van Doorslaer E, García-Gómez P, Van Ourti T: **Association between income and perinatal mortality in the Netherlands across gestational age**. *JAMA Network Open* 2021, **4**(11):e2132124-e2132124.
63. Zeng L, Yan H, Cheng Y, Dibley MJ: **Modifying effects of wealth on the response to nutrient supplementation in pregnancy on birth weight, duration of gestation and perinatal mortality in rural western China: double-blind cluster randomized controlled trial**. *International Journal of Epidemiology* 2011, **40**(2):350-362.
64. Kunkel M, Marete I, Cheng ER, Bucher S, Liechty E, Esamai F, Moore JL, McClure E, Vreeman RC: **Place of delivery and perinatal mortality in Kenya**. In: *Seminars in perinatology: 2019*: Elsevier; 2019: 252-259.
65. Ronsmans C, Chowdhury ME, Koblinsky M, Ahmed A: **Care seeking at time of childbirth, and maternal and perinatal mortality in Matlab, Bangladesh**. *Bulletin of the World Health Organization* 2010, **88**(4):289-296.
66. Khanam R, Baqui AH, Syed MIM, Harrison M, Begum N, Quaiyum A, Saha SK, Ahmed S: **Can facility delivery reduce the risk of intrapartum complications-related perinatal mortality? Findings from a cohort study**. *Journal of Global Health* 2018, **8**(1).

Table 1: Number of study participants included by country

Country	Weighted sample	Percentage (%)
Angola	13,276	4.23
Burkina Faso	12,210	3.89
Benin	13,542	4.31
Burundi	13,522	4.31
Cote d'Ivoire	9,690	3.08
Cameroon	10,024	3.19
Ethiopia	10,941	3.48
Gabon	6,045	1.92
Ghana	8,533	2.72
Gambia	7,608	2.42
Guinea	7,828	2.49
Kenya	17,316	5.51
Liberia	5,224	1.66
Lesotho	3,112	0.99
Madagascar	12,253	3.90
Malawi	34,499	10.98
Mozambique	5,491	1.75
Nigeria	34,018	10.83
Rwanda	8,267	2.63
Sierra Leone	9,718	3.09

Chad	18,635	5.93
Togo	6,706	2.14
Tanzania	10,806	3.44
Uganda	15,185	4.83
South Africa	3,541	1.13
Zambia	9,750	3.10
Zimbabwe	6,360	2.02
Overall	314,099	100

Table 2: Descriptive characteristics of women aged 15-49 in sub-Saharan African countries

**Variables**	**Weighted frequency**	**Percentage (%)**
**Residence**		
Rural	213,371	67.93
Urban	100,728	32.07
**Maternal age (in years)**		
15-24	91,416	29.10
25-34	149,661	47.65
≥35	73,021	23.25
**Maternal education**		
No education	110,470	35.17
Primary	109,700	34.93
Secondary	79,648	25.36

Higher	14,281	4.55
**Household wealth status**		
Poorest	71,982	22.92
Poorer	67,690	21.55
Middle	62,682	19.96
Richer	59,357	18.90
Richest	52,288	16.68
**Media exposure**		
No	121,460	38.67
Yes	192,639	61.33
**Parity**		
One birth	49,888	15.88
Two to four births	163,314	51.99
Five births and above	100,896	32.12
**Perceived distance to health facility**		
A big problem	144,906	46.13
Not a big problem	169,193	53.87
**Preceding birth interval**		
Primiparous	75,343	23.99
< 2 years	44,353	14.12
2 – 4 years	156,572	49.85
≥ 5 years	37,831	12.04
**Maternal working status**		

No	113,721	36.21
Yes	200,378	63.79
**Place of delivery**		
Home	118,609	37.76
Health facility	195,490	62.24
**ANC**		
No	123,981	39.47
Yes	190,118	60.53

Table 3: Multilevel binary logistic regression analysis of factors associated with perinatal mortality in SSA

Variables	Null model	Model II (AOR with 95% CI)	Model III (AOR with 95% CI)	Model IV (AOR with 95% CI)
**Maternal age (in years)**				
15-24		1		1
25-34		0.97 (0.92, 1.02)		0.96 (0.91, 1.01)
≥ 35		1.15 (1.07, 1.23)		1.13 (1.06, 1.21) *
**Maternal education status**				
No education		1		1
Primary		0.95 (0.91, 1.00)		1.05 (0.99, 1.10)
Secondary		0.89 (0.84, 0.95)		0.94 (0.89, 1.00)
Higher		0.79 (0.70, 0.89)		0.82 (0.73, 0.93) **

**Household wealth status**				
Poorest		1		1
Poorer		0.95 (0.90, 1.01)		0.94 (0.90, 1.00)
Middle		0.93 (0.88, 0.98)		0.93 (0.88, 0.98) **
Richer		0.91 (0.86, 0.97)		0.93 (0.87, 0.99) **
Richest		0.88 (0.82, 0.94)		0.92 (0.85, 1.00)
**Media exposure**				
No		1.16 (1.11, 1.21)		1.12 (1.08, 1.17) *
Yes		1		1
**Parity**				
< 2		1		1
2-4		1.34 (1.24, 1.45)		1.35 (1.25, 1.47) *
≥5		1.64 (1.49, 1.81)		1.69 (1.53, 1.86) **
**Preceding birth interval**				
< 2 years		1		1
2-4 years		0.71 (0.68, 0.75)		0.70 (0.67, 0.74) **
≥ 5 years		0.91 (0.85, 0.97)		0.91 (0.84, 0.97) *
Primiparous		1.20 (1.12, 1.30)		1.20 (1.12, 1.30)
**Place of delivery**				
Health facility delivery		1		1
Home delivery		1.10 (1.05, 1.15)		1.10 (1.06, 1.15) *
**ANC visit**				
No		1		1

Yes		0.66 (0.63, 0.69)		0.66 (0.63, 0.69) **
**Residence**				
Urban			1	1
Rural			1.14 (1.09, 1.19)	1.08 (1.02, 1.13) *
**SSA region**				
East Africa			1	1
West Africa			1.32 (1.27, 1.38)	1.30 (1.24, 1.36) **
Southern Africa			1.02 (0.89, 1.17)	1.11 (0.96, 1.27)
Central Africa			1.10 (1.05, 1.16)	1.05 (1.00, 1.11)
**Distance to health facility**				
Not big problem			1	1
Big problem			1.03 (0.99, 1.07)	1.06 (1.02, 1.10) *
**Random effect analysis results**				
LLR	-51098.94	-50466.46	-50998.78	-50390.07
Deviance	102197.9	100932.92	101997.6	100780.1

**ANC: Antenatal Care, AOR: Adjusted Odds Ratio, CI: Confidence Interval, LLR: Log-likelihood Ratio,* * $p < 0.05$, ** $p < 0.01$

Figure legend

Figure 1: Perinatal mortality rate across countries in SSA

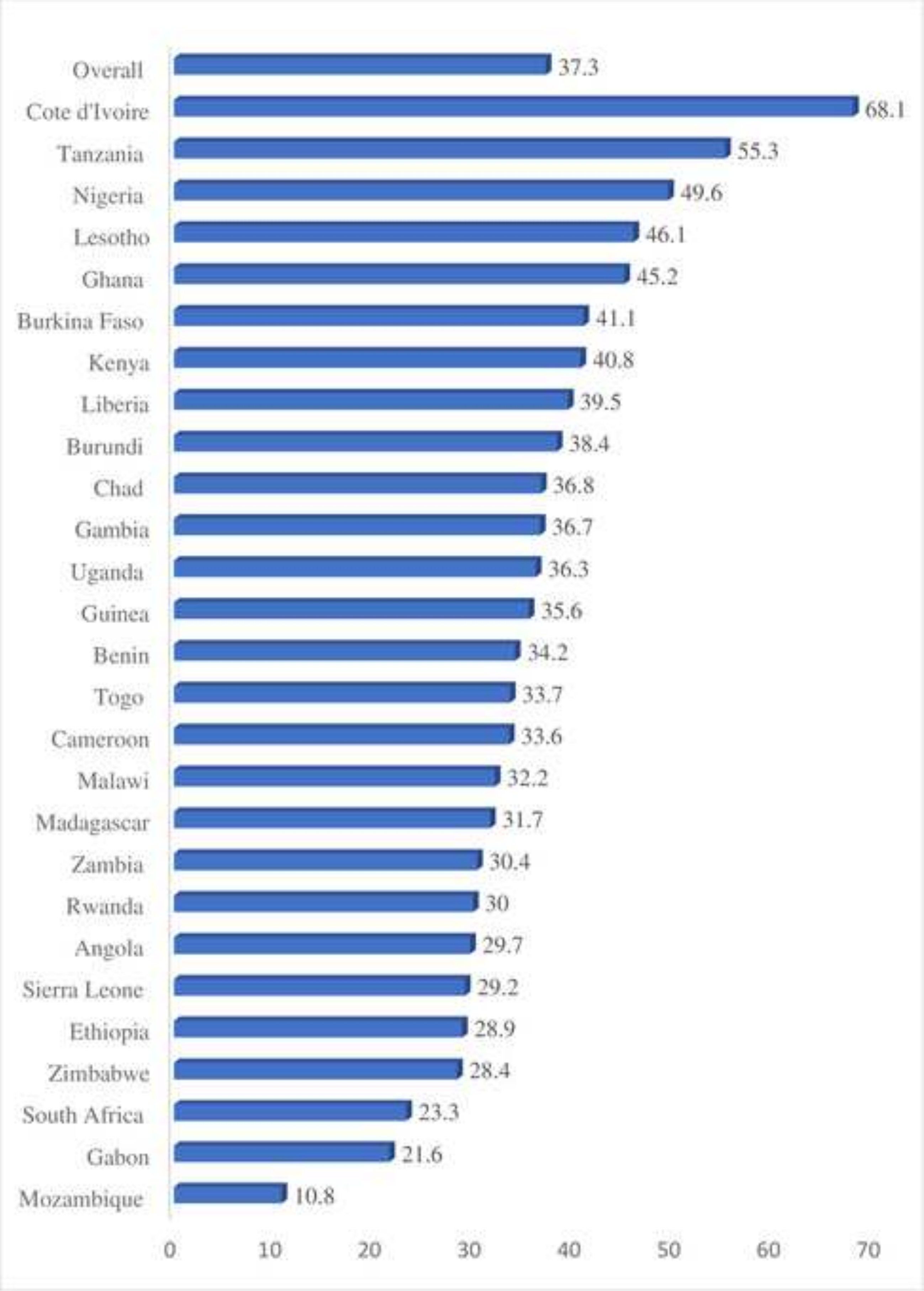

Figure 1: Perinatal mortality rate across countries in SSA

Factors associated with perinatal mortality in Sub-Saharan Africa: A multilevel analysis

Meklit Melaku Bezie[1], Hiwot Altaye Asebe[2], Angwach Abrham Asnake[3], Bezawit Melak Fente[4], Yohannes Mekuria Negussie[5], Zufan Alamrie Asmare[6], Mamaru Melkam[7], Beminate Lemma Seifu[8]

[1]Department of Public Health, Institute of Public Health, College of Medicine and Health Sciences and Comprehensive Specialized Hospital, University of Gondar, Gondar, Ethiopia

[2]Department of Public Health, College of Medicine and Health Sciences, Samara University, Samara, Ethiopia

[3]Department of Epidemiology and Biostatistics, School of Public Health, College of Medicine and Health Sciences, Wolaita Sodo University, Wolaita Sodo, Ethiopia

[4]Department of General Midwifery, School of Midwifery, College of Medicine & Health Sciences, University of Gondar, Gondar, Ethiopia

[5]Department of Medicine, Adama General Hospital and Medical College, Adama, Ethiopia

[6]Department of Ophthalmology, School of Medicine and Health Science, Debre Tabor University, Debre Tabor, Ethiopia.

[7]University of Gondar College of Medicine and Health Science, Department of Psychiatry, Gondar, Ethiopia

[8]Department of Public Health, College of Medicine and Health Sciences, Samara University, 21 Samara, Ethiopia

Corresponding Author: Meklit Melaku Bezie

Email: mesiyemaki@gmail.com

Abstract

Background: Perinatal mortality is a major global public health concern, especially in sub-Saharan Africa (SSA). Despite perinatal mortality ~~is~~being a major public health concern in SSA, there are very limited studies on the incidence and factors associated with perinatal mortality. Therefore, we aimed to investigate the factors associated with perinatal mortality in SSA.

Methods: A secondary data analysis was conducted based on the Demographic and Health Survey (DHS) data of 27 SSA countries. About 314,099 births in the preceding five years of the ~~survey was~~surveys were considered for the analysis. A multilevel binary logistic regression model was fitted to identify factors associated with perinatal mortality. Deviance (-2Log-Likelihood Ratio (LLR)) was used for model comparison. The Adjusted Odds Ratio (AOR) with the 5% Confidence Interval (CI) of the best-fitted model was used to verify the significant association between factors and perinatal mortality.

~~Result~~Results: The perinatal mortality rate in sub-Saharan Africa (SSA) was 37.31 per ~~1000~~1,000 births (95% CI: 36.65, 37.98). In the final best-~~fitted~~fit model, ~~individual level variables such as~~factors significantly associated with higher perinatal mortality included media exposure (AOR: 1.12, 95% CI: 1.08, 1.17), maternal age ≥ 35 years (AOR: 1.13, 95% CI: 1.06, 1.21), ~~preceding birth interval of 2 – 4 years (AOR: 0.70, 95% CI: 0.67, 0.74) and five years or above (AOR= 0.91, 95% CI: 0.84, 0.97),~~ health facility delivery (AOR~~=~~: 1.10, 95% CI: 1.06, 1.15), ~~Antenatal Care (ANC) (AOR = 0.66, 95% CI: 0.63, 0.69), having higher level of education (AOR = 0.82, 95% CI: 0.73, 0.93), belonged to the middle household wealth (AOR = 0.93, 95% CI: 0.88, 0.98) and richer household wealth (AOR = 0.93, 95% CI: 0.87, 0.99),~~ having 2-4 births (AOR~~ =~~: 1.35, 95% CI: 1.25, 1.47), five or ~~above~~more births (AOR~~ =~~: 1.69, 95% CI: 1.53, 1.86~~) were~~

Formatted: Pattern: Clear (White)

Formatted: Pattern: Clear (White)

Formatted: Pattern: Clear (White)

Formatted: Pattern: Clear (White)

Formatted: Pattern: Clear (White)

Formatted: Pattern: Clear (White)

Formatted: Pattern: Clear (White)

Formatted: Pattern: Clear (White)

Formatted: Pattern: Clear (White)

Formatted: Pattern: Clear (White)

~~significantly associated with perinatal mortality. In addition, community level variables such as belonged to~~), residence in West Africa (AOR~~ =~~: 1.30, 95% CI: 1.24, 1.36~~), central~~) or Central Africa (AOR~~ =~~: 1.05, 95% CI: 1.00, 1.11), rural ~~residents~~residency (AOR~~ =~~: 1.08, 95% CI: 1.02, 1.13~~)~~), and ~~big problem to access~~difficulty accessing a health facility (AOR~~ =~~: 1.06, 95% CI: 1.02, 1.10~~) were~~). In contrast, factors significantly associated with lower perinatal mortality~~.~~ were a preceding birth interval of 2-4 years (AOR: 0.70, 95% CI: 0.67, 0.74) or five or more years (AOR: 0.91, 95% CI: 0.84, 0.97), Antenatal Care (ANC) visit (AOR: 0.66, 95% CI: 0.63, 0.69), higher education levels (AOR: 0.82, 95% CI: 0.73, 0.93), middle household wealth (AOR: 0.93, 95% CI: 0.88, 0.98), and richer household wealth (AOR: 0.93, 95% CI: 0.87, 0.99)

Conclusion: Perinatal mortality was a major public health problem in SSA. Maternal socio-demographic, obstetrical, and healthcare-related factors are significantly associated with perinatal mortality. The findings of this study highlighted the need for holistic healthcare interventions targeting enhancing maternal healthcare services to reduce the incidence of perinatal mortality.

Keywords: Demographic and Health Survey, Perinatal mortality, Multilevel analysis, sub-Saharan Africa

Formatted: Pattern: Clear (White)
Formatted: Pattern: Clear (White)
Formatted: Pattern: Clear (White)
Formatted: Pattern: Clear (White)
Formatted: Pattern: Clear (White)
Formatted: Pattern: Clear (White)
Formatted: Pattern: Clear (White)
Formatted: Pattern: Clear (White)
Formatted: Pattern: Clear (White)
Formatted: Pattern: Clear (White)
Formatted: Pattern: Clear (White)
Formatted: Pattern: Clear (White)
Formatted: Tab stops: Not at 2.39"

Background

Perinatal mortality is defined as the death of a fetus after 28 weeks of gestation (stillbirth) plus the death of a neonate within 7 days of life [1]. Annually, over 6.9 million perinatal deaths occur globally [2-5], and almost all cases occur in Low-income and Middle-income Countries (LMICs) [5]. Despite the substantial reduction in global perinatal mortality [6], LMICs such as sub-Saharan African (SSA) countries continue sharing the huge burden of global perinatal deaths [7]. Annually, nearly 7 million perinatal deaths occur globally, including 3 to 4 million stillbirths and 3 million early neonatal deaths, with approximately 99% of these deaths taking place in low- and middle-income regions, predominantly in sub-Saharan Africa [8]

~~LMICs are still seemingly walking a patchy road towards achieving the Sustainable Development Goal (SDG), which is to end preventable deaths of under-five children in 2023 [8]. Of the global 6.9 million perinatal deaths, the vast majority (98%) of these deaths were reported to have occurred in SSA [9]. Over three-quarters of perinatal mortality is due to preventable causes [10, 11]. The perinatal mortality rate indicates the accessibility and quality of healthcare for the mother and baby [12]. For millions of families in low- and middle-income nations, perinatal mortality continues to be one of the most devastating pregnancy outcomes [13]. Currently, available evidence shows that perinatal mortality reflects the quality of maternal healthcare services during pregnancy, delivery, and postnatal care [14, 15]. It results from the complex interaction of individual-level factors linked to maternal lifestyle and obstetric complications, which could be exacerbated by underlying community-level factors such as poor access to quality maternal and newborn health services.~~

LMICs are still seemingly walking a patchy road towards achieving the Sustainable Development Goal (SDG), which is to end preventable deaths of under-five children in 2023 [9]. Of the global 6.9 million perinatal deaths, the vast majority (98%) of these deaths were reported to have occurred in SSA [10]. Over three-quarters of perinatal mortality is due to preventable causes [11, 12]. The perinatal mortality rate indicates the accessibility and quality of healthcare for the mother and baby [13]. For millions of families in low- and middle-income nations, perinatal mortality continues to be one of the most devastating pregnancy outcomes [14]. Currently, available evidence shows that perinatal mortality reflects the quality of maternal healthcare services during pregnancy, delivery, and postnatal care [15, 16]. It results from the complex interaction of individual-level factors linked to maternal lifestyle and obstetric complications, which could be exacerbated by underlying community-level factors such as poor access to quality maternal and newborn health services.

Perinatal mortality is a key indicator of women's health-seeking behavior, maternal healthcare access, socioeconomic status, and the standard of obstetric care ~~[16].~~[17]. Beyond losing the child, perinatal mortality causes emotional and psychological stress for the mother. Mother may also experience social stigma or strained relationships with their partners ~~[17].~~[18]. In addition, it has a significant financial cost, worsens the health of the mother, perpetuates the cycle of poverty, and impedes social and economic development, especially in Sub-Saharan Africa (SSA), where child mortality is eleven times higher than in developed nations ~~[18].~~[19].

Previous studies published on determinants of perinatal mortality revealed that maternal education ~~[19]~~[20], parity ~~[20]~~[21], residence ~~[21]~~[22], preceding birth interval ~~[22]~~[23], place of delivery ~~[23]~~[24], number of Antenatal Care (ANC) ~~[21]~~[22], media exposure ~~[24]~~[25] and wealth status were significantly associated with perinatal mortality. Several interventions ~~aimed at reducing~~ to lower the Perinatal Mortality Rate (PMR) have been ~~essential and~~ implemented ~~throughout the~~

Formatted: Font color: Auto, Pattern: Clear

Formatted: Font color: Auto, Pattern: Clear

Formatted: Font color: Auto, Pattern: Clear

~~world,~~globally, aiming to reduce ~~stillbirth~~stillbirths and ~~neonate death~~neonatal deaths to 12 per 1,000 ~~birth in~~births by 2030 ~~[25],~~ through the adoption of the Every Newborn Action Plan (ENAP) ~~[11, 26].~~[12, 26, 27]. Launched in 2014 by the World Health Organization (WHO) and the United Nations Children's Fund (UNICEF), ENAP seeks to prevent newborn deaths and stillbirths while improving maternal and child health worldwide. However, ~~progression of~~ PMR in ~~SSA~~sub-Saharan Africa remains alarmingly high compared to developed ~~countries~~nations [~~27~~28].

Despite ~~the~~ perinatal mortality ~~continues as~~continuing to be a major public health problem in SSA, there are limited published studies on the magnitude and factors associated with perinatal mortality in SSA. Therefore, we aimed to investigate the magnitude and factors associated with perinatal mortality in SSA based on Demographic and Health Survey (DHS) data from 27 SSA countries. This study will guide program planners and decision-makers ~~to design~~in designing evidence-based interventions to reduce ~~the incidence of~~ perinatal mortality in SSA.

Methods

Study design and sample

We analyzed DHS data from 27 sub-Saharan African countries. The DHS data is collected every five years to update basic health and health-related indicators of the country. It is assumed to be nationally representative, where a multi-stage stratified sampling is employed to recruit representative samples. The Enumeration Area (EA) ~~and household were used~~served as ~~a~~the primary ~~and~~ sampling unit, while households were the secondary sampling unit. ~~The birth record dataset was used to extract data on the~~Data on perinatal mortality and independent variables~~. All the important variables~~ were extracted from the Births Record (BR) dataset. Key variables from

Formatted: Font color: Auto, Pattern: Clear
Formatted: Font color: Auto, Pattern: Clear
Formatted: Font color: Auto, Pattern: Clear
Formatted: Font color: Auto, Pattern: Clear
Formatted: Font color: Auto, Pattern: Clear
Formatted: Font color: Auto, Pattern: Clear
Formatted: Font color: Auto
Formatted: Font color: Auto
Formatted: Font color: Auto
Formatted: Font color: Auto
Field Code Changed

27 sub-Saharan African (SSA) countries were collected and ~~appended together~~combined for the final analysis. ~~A~~ The dataset included a total of 314,099 births from women of reproductive~~-~~ age ~~women in~~within the five years preceding the survey ~~were used~~.

~~Dependent variable~~

~~The dependent variable for this study was perinatal mortality. Perinatal mortality was a composite of stillbirth and early neonatal death (deaths occurred before 7 days of birth) [28, 29]. The outcome variable (perinatal mortality) was binary and recoded as "yes" and "no".~~

Dependent variable

The dependent variable for this study was perinatal mortality. Perinatal mortality was a composite of stillbirth and early neonatal death (deaths occurred before 7 days of birth) [29, 30]. The outcome variable (Perinatal Mortality) was binary and recoded as "Yes" and "No".

Covariates

We used both individual- and community-level variables for our study. Maternal ~~age~~Age (grouped as 15 – 24, 25 – 34, and 35 -49 years), ~~media exposure~~Media Exposure (grouped as ~~no~~No and ~~yes), household wealth status~~Yes), Household Wealth Status (grouped as ~~poorest, poorer, middle, richer~~Poorest, Poorer, Middle, Richer, and ~~richest), preceding birth interval~~Richest), Preceding Birth Interval (grouped as < 2 years, 2 – 4 years, ≥5 years and ~~primiparous), place~~Primiparous), Place of ~~delivery~~Delivery (grouped as ~~home~~Home and ~~health facility), parity~~Health Facility), Parity (grouped as ~~one, two – four~~One, Two – Four and ~~five~~Five births or above), ANC visits (grouped as ~~no~~No and ~~yes), maternal educational status~~Yes), Maternal Educational Status (grouped as ~~no, primary, secondary~~No, Primary, Secondary and ~~higher education~~Higher

Education), and ~~maternal working status~~Maternal Working Status (grouped as ~~no~~No and ~~yes~~Yes) were the individual-level variables. Whereas, ~~residence~~Residence (grouped as ~~urban~~Urban and ~~rural), distance~~Rural), Distance from a ~~health facility~~Health Facility (grouped as a big problem and not a big problem), and SSA region (grouped as central, eastern, western, and southern regions) were considered as community-level variables.

Data management and analysis

To ensure the study results were representative, we used DHS sample weights to account for unequal probability sampling across strata and non-response. Descriptive characteristics including frequency and proportion were reported based on the weighted data to describe the study population. A multilevel binary logistic regression model was fitted to account for both the individual- and community-level variables. Stata version 17 was used for data management and analysis.

~~The~~A multilevel binary logistic regression model was ~~fitted due~~employed to account for the hierarchical structure of DHS data.~~-~~ The Intra-cluster Correlation Coefficient (ICC), Median Odds Ratio (MOR), and Likelihood Ratio (LR) ~~test were computed to assess the variability of perinatal mortality across clusters. Four models were fitted and deviance (-2Log likelihood Ratio (-2LLR)).~~tests were calculated to evaluate the variability of perinatal mortality across clusters. Four models were fitted, and model comparison was conducted using deviance (-2 Log-likelihood Ratio (-2LLR)), with the lowest value indicating the best-fitting model for the data. Deviance, expressed as the -2 Log-likelihood Ratio (-2LLR), is a statistical metric used to compare nested models in logistic regression and other likelihood-based approaches [31]. For nested models, it assesses whether the more complex model provides a better fit to the data than a simpler model by

determining if the additional parameters in the complex model significantly enhance its performance. When it approaches zero, it indicates that the model fits the data exceptionally well. Model I was the null model fitted without covariates, Model II was the model with individual-level variables, Model 3 was the model with community-level variables and Model IV was the model with both individual and community-level variables. Variables with a P-value ≤ 0.2 in the bivariable analysis were selected for multivariable multilevel logistic regression analysis. In the final best-fitted model, the Adjusted Odds Ratio (AOR) with 95% Confidence Interval (CI) was used to establish the statistical significance and strength of the association.

Ethical consideration

~~In the case of~~ For this study, we have ~~been granted~~received an ~~authorized~~authorization letter from the ~~measure~~Measure DHS program for using the data. DHS ~~is~~provides publicly available de-identified data~~;~~, so ethical approval is not needed. DHS employs several approaches to de-identify the data to ensure the privacy and confidentiality of respondents, such as data anonymization and the removal of personal identifiers.

Results

Descriptive characteristics of study participants

A total weighted sample of 314,099 births in the last five years preceding the survey was included~~.~~ (Table 1). More than two-thirds (68%) of participants ~~were~~ resided in rural areas. About 110,470 (35.17%) and 109,700 (35%) of the mothers didn’t have formal education and primary education, respectively. Approximately half of the mothers (47.65%) were within the age range of 25-34 years. Nearly one-fourth (22.9%) and 16.8% of participants belonged to the poorest and richest

households, respectively. Only 13.33% of mothers had a history of terminating a pregnancy (Table 2).

The perinatal mortality rate in SSA

The perinatal mortality rate in SSA was 37.31 per 1000 births (95% CI: 36.65, 37.98). It has varied across countries ranging from the lowest in Mozambique (10.8 per 1000 births) to the highest in Cote D'Ivoire (68.1 per 1000 births) (Figure 1).

Factors associated with perinatal mortality

Random effect analysis results

We examined whether the multilevel binary logistic regression model was significant over the single-level binary logistic regression model using the Likelihood Ratio (LR) test. The LR test was statistically significant ($p<0.05$), indicating that the multilevel binary logistic regression model was best fitted to the single-level binary logistic regression analysis. Model comparison was employed by using deviance since models are nested models. Four models (mode I: 102197.9, model II: 100932, model III: 101997.6, and model IV: 100780.1) were fitted and model IV value (100780.1) was selected as the best-fitted model to explain perinatal mortality in SSA since it had the lowest deviance value. The MOR was 1.24 showing that if a mother of a child moved from a cluster with a low risk of perinatal mortality to a cluster with a high risk, the odds of experiencing perinatal mortality increased by 1.24 times.

Fixed effect analysis results

In multivariable multilevel logistic regression analysis;, individual-level variables such as maternal age, maternal education, parity, ANC, preceding birth interval, and place of delivery, and

community-level variables such as residence, distance to the health facility, and sub-Saharan African region had a statistically significant association with perinatal mortality.

The odds of perinatal mortality of babies to mothers aged 35 – 49 years were 1.13 times (AOR = 1.13, 95% CI: 1.06, 1.21) higher compared to those born to mothers aged 15-24 years. The odds of experiencing perinatal mortality among mothers who had higher education were decreased by 18% (AOR = 0.82, 95% CI: 0.73, 0.93) compared to mothers who had no formal education. The odds of perinatal mortality among mothers who belonged to middle and richer household wealth were decreased by 7% (AOR = 0.93, 95% CI: 0.88, 0.98) and 7% (AOR = 0.93, 95% CI: 0.87, 0.99) compared to those belonged to the poorest household, respectively. Mothers who had no media exposure had 1.12 times (AOR = 1.12, 95% CI: 1.08, 1.17) higher odds of perinatal mortality compared to those who had media exposure. Multiparous and grand multiparous mothers had 1.35 times (AOR = 1.35, 95% CI: 1.25, 1.47) and 1.69 times (AOR = 1.69, 95% CI: 1.53, 1.86) higher odds of perinatal mortality compared to primiparous mother, respectively.

The odds of perinatal mortality among mothers who had preceding birth interval of 2 – 4 years and 5 years or above were decreased by 30% (AOR = 0.70, 95% CI: 0.67, 0.74) and 9% (AOR = 0.91, 95% CI: 0.84, 0.97) compared to those who had birth interval of less than 2 years, respectively. Mothers who gave birth at a health facility had 1.10 times (AOR = 1.10, 95% CI: 1.06, 1.15) higher odds of perinatal mortality than those who gave birth at home. The odds of perinatal mortality among mothers who had ANC visits were decreased by 34% (AOR = 0.66, 95% CI: 0.63, 0.69). Being a rural resident increased the odds of perinatal mortality by 8 % (AOR = 1.08, 95% CI: 1.02, 1.13). Mothers in West Africa and Central Africa had 1.30 times (AOR = 1.30, 95% CI: 1.24, 1.36) and 1.05 times (AOR = 1.05, 95% CI: 1.00, 1.11) higher odds of perinatal mortality compared to

mothers in East Africa, respectively. Mothers who had big problems accessing health care increased the odds of perinatal mortality by 6% (AOR = 1.06, 95% CI: 1.02, 1.10) (Table 3).

Discussion

In our study, the perinatal mortality rate in SSA was found to be 37 per 1000 births which varied across regions. It was higher than a study reported in Indonesia [30][32] and India [31].[33]. This difference couldmay be because sub-Saharan African countries have been facingdue to the extensive socio-political and economic crises attributable tothat sub-Saharan African countries have been experiencing, which are caused by both man-made and natural disasters that can influencecontribute to increased perinatal mortality [32].[34]. The findings of our study demonstrated that perinatal mortality in SSA remains a significant public health problem. The higher perinatal mortality rate is the result of a multiplicity of factors such as limited access to ANC, lack of quality of care during delivery, higher prevalence of poverty, low level of maternal education, maternal nutritional deficiency, teenage pregnancy, high prevalence of maternal infections (HIV, malaria, syphilis…etc), preterm births, low birth weight, and home delivery [33, 34]. Besides[35, 36]. Additionally, in many African countries, some deep-rootedentrenched cultural beliefs and practices influenceaffect maternal and neonatal care, such as the delayed initiation of breastfeeding, and other harmful traditional remedies which influencewhich can impact perinatal outcomes [3537].

Field Code Changed

In the multilevel analysis variables such as media exposure, maternal age, parity, household wealth status, preceding birth interval, maternal education, place of delivery, ANC, distance to health facility, residence, and SSA region were significantly associated with perinatal mortality. The odds of perinatal mortality among mothers aged 35 years and above were higher compared to those aged

15-24 years. This finding ~~agreed~~is consistent with previous ~~study findings [36, 37], this could be~~ studies [38, 39], likely due to the strong association between advanced maternal age ~~being strongly associated with~~ and an ~~increased~~elevated risk of adverse pregnancy conditions~~ such as~~. These conditions include pregnancy-induced hypertension, antepartum hemorrhage, cesarean delivery, preterm delivery, low birth weight, and premature rupture of ~~the membrane~~ membranes, all of which ~~in turn~~can increase the risk of perinatal deaths ~~[38, 39].~~[40, 41]. Maternal education reduces the risk of perinatal mortality. This finding was in line with studies reported in Nigeria ~~[40]~~[42], Spain ~~[41]~~[43], and Pakistan ~~[42].~~[44]. It could be due to maternal education plays a significant role in improving maternal and perinatal health outcomes ~~[43].~~[45]. Educated mothers understand health-related information, are more likely to seek perinatal care, recognize danger signs of pregnancy, and take timely interventions ~~[44].~~[46]. In addition, educated mothers are highly likely to attend prenatal care early and regularly as well as more likely to give birth in healthcare facilities. Furthermore, mothers with higher education are empowered to make their own decisions about their health and childbirth.

Formatted: Font: Calibri, 11 pt

The odds of perinatal mortality are high among mothers with five or more children compared to babies born to mothers who have fewer than two children. ~~This study is in line with previous findings in Australia which stated that grand multi-para mothers are at risk of perinatal [45, 46]. This could be due to multipara mothers may prevail to uterine and placenta changes with each pregnancy which predispose to adverse pregnancy and perinatal outcomes and poor nutritional status of the mother which lead to low birth weight in turn results in stillbirth and multiparous mother with low resource setting might face financial constraint that creates a barrier to quality health service. As the birth interval increases, it is significantly associated with lower perinatal deaths, which is consistent with the study done in Bangladesh [47] and Ethiopia[22]. The reason~~

~~might be longer birth intervals give the mother time to recover physically and psychologically and it helps in nutritional recovery which strengthens the mother's immunity system in turn helps to avoid maternal infection as well as the fetus [48]~~This study is in line with previous findings in Australia which stated that grand multiparous mothers are at risk of perinatal [47, 48]. This may be due to multiparous mothers undergoing uterine and placental changes that increase the risk of adverse outcomes. Poor maternal nutrition can lead to low birth weight and higher stillbirth risk. Additionally, financial constraints in low-resource settings may limit access to quality health services for these mothers.~~. ANC reduces the risk of perinatal mortality. This finding is consistent with the study findings in Ethiopia [49, 50], and Gambia [51]. The possible explanation might be due to ANC makes it possible to track foetal development in order to provide timely interventions and preventive measures, as well as to diagnose pregnancy complications such as gestational diabetes and preeclampsia early on [52]. It creates the opportunity to connects mothers with health professionals, helping them prepare for birth and emergencies [53]. Our study revealed that perinatal mortality was significantly higher in rural areas. This is consistent with the study done in India [54]. Mothers could rely on traditional birth attendants because living in a rural area may make it more likely that there won't be enough qualified birth attendants, which causes people to be unaware of the risks associated with pregnancy and prenatal care [55].~~

As the birth interval increases, it is significantly associated with lower perinatal deaths, which is consistent with studies in Bangladesh [49] and Ethiopia[23]. It could be due to having a longer preceding birth interval enable the mother to recover physically and psychologically, and helps their nutritional recovery which strengthens the mother's immunity system, which in turn helps to avoid maternal infection as well as that of the fetus [50]. ANC reduces the risk of perinatal mortality. This finding is consistent with the study findings in Ethiopia [51, 52], and Gambia [53].

The possible explanation might be due to ANC makes it possible to track fetal development in order to provide timely interventions and preventive measures, as well as to diagnose pregnancy complications such as gestational diabetes and preeclampsia early on [54]. It creates the opportunity to connect mothers with health professionals, helping them prepare for birth and any emergencies [55]. Our study revealed that perinatal mortality was significantly higher in rural areas. Mothers who had big problems in accessing healthcare had higher odds of perinatal mortality. This study is in line with Ethiopia [56, 57]. The rise in missed appointments and the postponed start of maternity care could serve as justification for this [58]. Furthermore, it can be challenging to promptly address complications like hemorrhage and obstructed labour in low socioeconomic households where transportation is a major challenge [59].

According to our findings, higher socio-economic class is associated with lower perinatal mortality. This is supported by a study done in the Netherlands [60], and in western China [61]. Our study states that babies born in health facilities had an increased risk of perinatal mortality as compared to babies born at home. This study supported the study done in Kenya [62]. This may be explained by the fact that babies born in health facilities often come from mothers who are already triaged as high-risk, inherently increasing the perinatal death rate compared to mothers with uncomplicated pregnancies who deliver at home. Additionally, mothers who arrive at health facilities at the last minute due to emergencies may experience complications exacerbated by transportation delays [63, 64], and especially in SSA overcrowded facilities and resource constraints like lack of equipment, and skilled personnel further underestimate the effectiveness of health facilities.

This is consistent with the study done in India [56]. This could be due to the majority of rural mothers giving birth at home, so they are at higher risk of intrapartum and early postpartum complications [57].

Mothers who had big problems accessing healthcare had higher odds of perinatal mortality. This study is in line with findings in Ethiopia [58, 59]. The rise in missed appointments and the postponed start of maternity care could serve as justification for this [60]. Furthermore, it can be challenging to promptly address complications like hemorrhage and obstructed labor in low socioeconomic households where transportation is a major challenge [61].

According to our findings, higher socio-economic class is associated with lower perinatal mortality. This is supported by a study done in the Netherlands [62], and in western China [63]. Our study states that babies born in health facilities had an increased risk of perinatal mortality as compared to babies born at home. This study supported the study done in Kenya [64]. This may be explained by the fact that babies born in health facilities often come from mothers who are already triaged as high-risk, inherently increasing the perinatal death rate compared to mothers with uncomplicated pregnancies who deliver at home. Additionally, usually due to transportation and other related constraints mothers in SSA mothers who arrive at health facilities [65, 66], which in turn made them at increased risk of complications such as fetal distress, meconium aspiration, .etc.

Strengths and limitations of the study

The main strength of ~~the~~this study~~, it~~ is the use used ~~nationality~~ representative data from DHS of 27 sub-Saharan African countries, which involves a large sample size. This study also employed a multilevel technique that considers the hierarchical nature of the survey and therefore, the results

are reliable and robust. However, the data is cross-sectional, and the temporal relationship of causations cannot be established. This study might have a recall bias, for variables such as preceding birth, postnatal care, and antenatal care visits~~.~~, which should be taken into account when interpreting our results.

Conclusion

Perinatal mortality remains a significant public health concern in SSA. Advanced maternal age, high parity, being rural, having no media exposure, home delivery, and a big problem accessing health care ~~was~~were significantly associated with higher odds of perinatal mortality, while ANC, higher maternal education, belonging to a wealthier household, optimal and long preceding birth interval were significantly associated with lower odds of perinatal mortality. The findings of this study highlighted the need to enhance maternal education, ANC use, health facility delivery, and media exposure to reduce the incidence of perinatal mortality.

Abbreviations

ANC: Antenatal Care; AOR: Adjusted Odds Ratio; CI: Confidence interval; DHS: Demographic and Health Survey; EA: Enumeration Area; PSU: Primary Sampling Unit WHO: World Health Organization: SSA: Sub-Saharan Africa

~~Acknowledgements~~

Acknowledgments

We acknowledge the MEASURE DHS program for permitting the use of the dataset.

Authors' contributions

Conceptualization: MMB, HAA, AAA, BMF, YMN, ZAA, MM and BLS

Data curation: MMB, HAA, AAA, BMF, YMN, ZAA, MM and BLS

Formal analysis: MMB, HAA, AAA, BMF, YMN, ZAA, MM and BLS

Funding acquisition: MMB, HAA, AAA, BMF, YMN, ZAA, MM and BLS

Investigation: MMB

Methodology: MMB, HAA, AAA, BMF, YMN, ZAA, MM and BLS

Project administration: MMB, HAA, AAA, BMF, YMN, ZAA, MM and BLS

Resources: MMB, HAA, AAA, BMF, YMN, ZAA, MM and BLS

Software: MMB

Supervision: MMB, HAA, AAA, BMF, YMN, ZAA, MM and BLS

Validation: MMB, HAA, AAA, BMF, YMN, ZAA, MM and BLS

Visualization: MMB, HAA, AAA, BMF, YMN, ZAA, MM and BLS

Writing – original draft: MMB

Writing – review & editing: MMB, HAA, AAA, BMF, YMN, ZAA, MM and BLS. All authors critically reviewed, and revised the article, and gave final approval for publication.

References

1. Organization WH: **Neonatal and perinatal mortality: country, regional and global estimates**: World Health Organization; 2006.
2. Berhan Y, Berhan A: **Perinatal mortality trends in Ethiopia**. *Ethiopian journal of health sciences* 2014, **24**:29-40.
3. Organization WH: **Every newborn: an action plan to end preventable deaths**. 2014.
4. Hug L, Mishra A, Lee S, You D, Moran A, Strong KL, Cao B: **A neglected tragedy the global burden of stillbirths: report of the UN inter-agency group for child mortality estimation, 2020**. In.: United Nations Children's Fund; 2020.
5. Hussain S: **Neonatal morbidity and mortality pattern in a tertiary care neonatal unit of a teaching hospital**. *Ann Pak Inst Med Sci* 2014, **10**(1):7-11.
6. Hug L, You D, Blencowe H, Mishra A, Wang Z, Fix MJ, Wakefield J, Moran AC, Gaigbe-Togbe V, Suzuki E: **Global, regional, and national estimates and trends in stillbirths from 2000 to 2019: a systematic assessment**. *The Lancet* 2021, **398**(10302):772-785.
7. Smith AC, Mutangiri W, Fox R, Crofts JF: **Millennium Development Goal 4: reducing perinatal and neonatal mortality in low-resource settings**. *Obstetrician & Gynaecologist* 2014, **16**(1).

~~8~~8. Ogunlesi TA, Adetoro OO: **Preventing Perinatal Mortality in the Developing Countries**. *Contemporary Obstetrics and Gynecology for Developing Countries* 2021:23-34.

9. Bossman E: **The post-2015 development agenda: progress towards sustainable development goal target on maternal mortality and child mortality in limited resource settings with mHealth interventions: a systematic review in Sub-Saharan Africa and Southern Asia**. UiT Norges arktiske universitet; 2019.

~~9~~10. Aminu M, Bar-Zeev S, White S, Mathai M, van Den Broek N: **Understanding cause of stillbirth: a prospective observational multi-country study from sub-Saharan Africa**. *BMC pregnancy and childbirth* 2019, **19**:1-10.

~~10~~11. Bhutta ZA, Das JK, Bahl R, Lawn JE, Salam RA, Paul VK, Sankar MJ, Blencowe H, Rizvi A, Chou VB: **Can available interventions end preventable deaths in mothers, newborn babies, and stillbirths, and at what cost?** *The Lancet* 2014, **384**(9940):347-370.

~~11~~12. Organization WH: **Reaching the every newborn national 2020 milestones: country progress, plans and moving forward**. 2017.

~~12~~13. Santosh A, Zunjarwad G, Hamdi I, Al-Nabhani JA, Sherkawy BE, Al-Busaidi IH: **Perinatal mortality rate as a quality indicator of healthcare in Al-Dakhiliyah Region, Oman**. *Sultan Qaboos University Medical Journal* 2013, **13**(4):545.

~~13~~14. Karlsen S, Say L, Souza J-P, Hogue CJ, Calles DL, Gülmezoglu AM, Raine R: **The relationship between maternal education and mortality among women giving birth in health care institutions: analysis of the cross sectional WHO Global Survey on Maternal and Perinatal Health**. *BMC public health* 2011, **11**:1-10.

~~14~~15. Beksinska M, Kunene B, Mullick S: **Maternal care: antenatal, peri and postnatal: women's health**. *South African health review* 2006, **2006**(1):297-314.

~~15~~16. Tunçalp Ö, Were W, MacLennan C, Oladapo O, Gülmezoglu A, Bahl R, Daelmans B, Mathai M, Say L, Kristensen F: **Quality of care for pregnant women and newborns—the WHO vision**. *Bjog* 2015, **122**(8):1045.

~~16~~17. Schmiegelow C, Minja D, Oesterholt M, Pehrson C, Suhrs HE, Boström S, Lemnge M, Magistrado P, Rasch V, Lusingu J: **Factors associated with and causes of perinatal mortality in northeastern Tanzania**. *Acta obstetricia et gynecologica Scandinavica* 2012, **91**(9):1061-1068.

~~17~~18. Fernández-Sola C, Camacho-Ávila M, Hernández-Padilla JM, Fernández-Medina IM, Jiménez-López FR, Hernández-Sánchez E, Conesa-Ferrer MB, Granero-Molina J: **Impact of perinatal death**

on the social and family context of the parents. *International journal of environmental research and public health* 2020, **17**(10):3421.

~~18~~19. Schwartz DA: **Challenges in improvement of perinatal health in developing nations: Role of perinatal pathology**. *Archives of Pathology and Laboratory Medicine* 2013, **137**(6):742-746.

~~19~~20. Tesema GA, Worku MG: **Individual-and community-level determinants of neonatal mortality in the emerging regions of Ethiopia: a multilevel mixed-effect analysis**. *BMC pregnancy and childbirth* 2021, **21**:1-11.

~~20~~21. Yirgu R, Molla M, Sibley L, Gebremariam A: **Perinatal mortality magnitude, determinants and causes in west Gojam: population-based nested case-control study**. *PloS one* 2016, **11**(7):e0159390.

~~21~~22. Kibria GMA, Burrowes V, Choudhury A, Sharmeen A, Ghosh S, Mahmud A, Kc A: **Determinants of early neonatal mortality in Afghanistan: an analysis of the Demographic and Health Survey 2015**. *Globalization and health* 2018, **14**:1-12.

~~22~~23. Jena BH, Biks GA, Gelaye KA, Gete YK: **Magnitude and trend of perinatal mortality and its relationship with inter-pregnancy interval in Ethiopia: a systematic review and meta-analysis**. *BMC pregnancy and childbirth* 2020, **20**:1-13.

~~23~~24. Nankabirwa V, Tumwine JK, Tylleskär T, Nankunda J, Sommerfelt H, Consortium PER: **Perinatal mortality in eastern Uganda: a community based prospective cohort study**. *PloS one* 2011, **6**(5):e19674.

~~24~~25. Andargie G, Berhane Y, Worku A, Kebede Y: **Predictors of perinatal mortality in rural population of Northwest Ethiopia: a prospective longitudinal study**. *BMC public health* 2013, **13**:1-7.

~~25~~26. Organization WH: **Every newborn action plan: progress report, May 2015**. 2015.

27. Plan ENA: **Every newborn: an action plan to end preventable deaths**. *Every Newborn Action Plan* 2014.

28~~26. Organization WH: **Every newborn action plan: progress report, May 2015**. 2015.~~

~~27~~. Wang H, Bhutta ZA, Coates MM, Coggeshall M, Dandona L, Diallo K, Franca EB, Fraser M, Fullman N, Gething PW: **Global, regional, national, and selected subnational levels of stillbirths, neonatal, infant, and under-5 mortality, 1980–2015: a systematic analysis for the Global Burden of Disease Study 2015**. *The Lancet* 2016, **388**(10053):1725-1774.

~~28~~29. Smith GC, Fretts RC: **Stillbirth**. *The Lancet* 2007, **370**(9600):1715-1725.

~~29~~30. Lehtonen L, Gimeno A, Parra-Llorca A, Vento M: **Early neonatal death: a challenge worldwide**. In: *Seminars in Fetal and Neonatal Medicine: 2017*: Elsevier; 2017: 153-160.

~~30~~31. Quintero A, Lesaffre E: **Comparing hierarchical models via the marginalized deviance information criterion**. *Statistics in Medicine* 2018, **37**(16):2440-2454.

32. Sariunita N, Idris H: **Regional perinatal mortality differences in Indonesia: Evidence from Indonesian demographic health survey**. *Public Health in Practice* 2024, **7**:100501.

~~31~~33. Misra A, Menon G, Sinha AP, Singh S, Rao MVV, Sharma S: **Risk factors for perinatal mortality in India: a systematic review of observational studies**. *International Journal of Community Medicine and Public Health* 2022, **9**(10):3849.

~~32~~34. Wise PH, Darmstadt GL: **Confronting stillbirths and newborn deaths in areas of conflict and political instability: a neglected global imperative**. *Paediatrics and International Child Health* 2015, **35**(3):220-226.

~~33~~35. Pervin J, Moran A, Rahman M, Razzaque A, Sibley L, Streatfield PK, Reichenbach LJ, Koblinsky M, Hruschka D, Rahman A: **Association of antenatal care with facility delivery and perinatal survival–a population-based study in Bangladesh**. *BMC pregnancy and childbirth* 2012, **12**:1-12.

~~34~~36. Ntambue AM, Malonga FK, Dramaix-Wilmet M, Ngatu RN, Donnen P: **Better than nothing? maternal, newborn, and child health services and perinatal mortality, Lubumbashi, democratic republic of the Congo: a cohort study**. *BMC pregnancy and childbirth* 2016, **16**:1-12.

~~35~~37. Cham M, Sundby J, Vangen S: **Maternal mortality in the rural Gambia, a qualitative study on access to emergency obstetric care**. *Reproductive health* 2005, **2**:1-8.

~~36~~38. Mutz-Dehbalaie I, Scheier M, Jerabek-Klestil S, Brantner C, Windbichler GH, Leitner H, Egle D, Ramoni A, Oberaigner W: **Perinatal mortality and advanced maternal age**. *Gynecologic and obstetric investigation* 2014, **77**(1):50-57.

~~37~~39. Salem Yaniv S, Levy A, Wiznitzer A, Holcberg G, Mazor M, Sheiner E: **A significant linear association exists between advanced maternal age and adverse perinatal outcome**. *Archives of gynecology and obstetrics* 2011, **283**:755-759.

~~38~~40. Saccone G, Gragnano E, Ilardi B, Marrone V, Strina I, Venturella R, Berghella V, Zullo F: **Maternal and perinatal complications according to maternal age: A systematic review and meta-analysis**. *International Journal of Gynecology & Obstetrics* 2022, **159**(1):43-55.

~~39~~41. Jacobsson B, Ladfors L, Milsom I: **Advanced maternal age and adverse perinatal outcome**. *Obstetrics & Gynecology* 2004, **104**(4):727-733.

~~40~~42. Fawole AO, Shah A, Tongo O, Dara K, El-Ladan AM, Umezulike AC, Alu FE, Eniayewun AB, Fabanwo AO, Adewunmi AA: **Determinants of perinatal mortality in Nigeria**. *International Journal of Gynecology & Obstetrics* 2011, **114**(1):37-42.

~~41~~43. Juárez S, Revuelta-Eugercios BA, Ramiro-Fariñas D, Viciana-Fernández F: **Maternal education and perinatal outcomes among Spanish women residing in southern Spain (2001–2011)**. *Maternal and child health journal* 2014, **18**:1814-1822.

~~42~~44. Fikree FF, Gray RH: **Demographic survey of the level and determinants of perinatal mortality in Karachi, Pakistan**. *Paediatric and perinatal epidemiology* 1996, **10**(1):86-96.

~~43~~45. Elo IT: **Utilization of maternal health-care services in Peru: the role of women's education**. *Health transition review* 1992:49-69.

~~44~~46. Zaki A, Fouad S: **Assessment of knowledge and practices of pregnant women toward danger signs of pregnancy**. *Mansoura Nursing Journal* 2021, **8**(1):13-32.

~~45~~47. Bai J, Wong FW, Bauman A, Mohsin M: **Parity and pregnancy outcomes**. *American journal of obstetrics and gynecology* 2002, **186**(2):274-278.

~~46~~48. Seyoum E, Bekele A, Tsegaye AT, Birhanu S: **Magnitude and determinants of adverse perinatal outcomes in Tefera Hailu Memorial Hospital, Sekota Town, northern Ethiopia**. *Global pediatric health* 2021, **8**:2333794X211015524.

~~47~~49. Hossain MB, Kanti Mistry S, Mohsin M, Rahaman Khan MH: **Trends and determinants of perinatal mortality in Bangladesh**. *PloS one* 2019, **14**(8):e0221503.

~~48~~50. Fortney JA, Higgins JE: **The effect of birth interval on perinatal survival and birth weight**. *Public Health* 1984, **98**(2):73-83.

~~49~~51. Shiferaw K, Mengiste B, Gobena T, Dheresa M: **The effect of antenatal care on perinatal outcomes in Ethiopia: A systematic review and meta-analysis**. *PloS one* 2021, **16**(1):e0245003.

~~50~~52. Dessu S, Dawit Z: **Perinatal mortality and associated factors among antenatal care attended pregnant mothers at public hospitals in Gamo Zone, Southern Ethiopia**. *Frontiers in Pediatrics* 2020, **8**:586747.

~~51~~53. Kumar S, Suso A, Wood E: **Effect of antenatal care on birth outcomes in The Gambia: a propensity score matching analysis**. 2024.

~~52~~54. Organization WH: **WHO recommendations on antenatal care for a positive pregnancy experience: summary: highlights and key messages from the World Health Organization's 2016 global recommendations for routine antenatal care**. In.: World Health Organization; 2018.

~~53~~55. Vogel JP, Habib NA, Souza JP, Gülmezoglu AM, Dowswell T, Carroli G, Baaqeel HS, Lumbiganon P, Piaggio G, Oladapo OT: **Antenatal care packages with reduced visits and perinatal mortality: a secondary analysis of the WHO Antenatal Care Trial**. *Reproductive health* 2013, **10**:1-7.

~~54~~56. Tayade Surekha A, Kumar N: **Socio demographic factors affecting perinatal mortality-A study in a rural setup**. *Parity* 2012, **11**(5.97).

~~55~~57. Thomas SL, Wakerman J, Humphreys JS: **Ensuring equity of access to primary health care in rural and remote Australia-what core services should be locally available?** *International journal for equity in health* 2015, **14**:1-8.

~~56~~58. Kibret GD, Demant D, Hayen A: **The effect of distance to health facility on neonatal mortality in Ethiopia**. *BMC Health Services Research* 2023, **23**(1):114.

~~57~~59. Målqvist M, Sohel N, Do TT, Eriksson L, Persson L-Å: **Distance decay in delivery care utilisation associated with neonatal mortality. A case referent study in northern Vietnam**. *BMC Public Health* 2010, **10**:1-9.

~~58~~60. Shukla VV, Eggleston B, Ambalavanan N, McClure EM, Mwenechanya M, Chomba E, Bose C, Bauserman M, Tshefu A, Goudar SS: **Predictive modeling for perinatal mortality in resource-limited settings**. *JAMA network open* 2020, **3**(11):e2026750-e2026750.

~~59~~61. Abu-Ghanem S, Sheiner E, Sherf M, Wiznitzer A, Sergienko R, Shoham-Vardi I: **Lack of prenatal care in a traditional community: trends and perinatal outcomes**. *Archives of gynecology and obstetrics* 2012, **285**:1237-1242.

~~60~~62. Vidiella-Martin J, Been JV, Van Doorslaer E, García-Gómez P, Van Ourti T: **Association between income and perinatal mortality in the Netherlands across gestational age**. *JAMA Network Open* 2021, **4**(11):e2132124-e2132124.

~~61~~63. Zeng L, Yan H, Cheng Y, Dibley MJ: **Modifying effects of wealth on the response to nutrient supplementation in pregnancy on birth weight, duration of gestation and perinatal mortality in rural western China: double-blind cluster randomized controlled trial**. *International Journal of Epidemiology* 2011, **40**(2):350-362.

~~62~~64. Kunkel M, Marete I, Cheng ER, Bucher S, Liechty E, Esamai F, Moore JL, McClure E, Vreeman RC: **Place of delivery and perinatal mortality in Kenya**. In: *Seminars in perinatology: 2019*: Elsevier; 2019: 252-259.

~~63~~65. Ronsmans C, Chowdhury ME, Koblinsky M, Ahmed A: **Care seeking at time of childbirth, and maternal and perinatal mortality in Matlab, Bangladesh**. *Bulletin of the World Health Organization* 2010, **88**(4):289-296.

~~64~~66. Khanam R, Baqui AH, Syed MIM, Harrison M, Begum N, Quaiyum A, Saha SK, Ahmed S: **Can facility delivery reduce the risk of intrapartum complications-related perinatal mortality? Findings from a cohort study**. *Journal of Global Health* 2018, **8**(1).

Table 1: Number of study participants included by country

Country	Weighted sample	Percentage (%)
Angola	13,276	4.23
Burkina Faso	12,210	3.89
Benin	13,542	4.31
Burundi	13,522	4.31
Cote d'Ivoire	9,690	3.08
Cameroon	10,024	3.19
Ethiopia	10,941	3.48
Gabon	6,045	1.92
Ghana	8,533	2.72
Gambia	7,608	2.42
Guinea	7,828	2.49
Kenya	17,316	5.51
Liberia	5,224	1.66
Lesotho	3,112	0.99
Madagascar	12,253	3.90
Malawi	34,499	10.98
Mozambique	5,491	1.75
Nigeria	34,018	10.83
Rwanda	8,267	2.63
Sierra Leone	9,718	3.09

Chad	18,635	5.93
Togo	6,706	2.14
Tanzania	10,806	3.44
Uganda	15,185	4.83
South Africa	3,541	1.13
Zambia	9,750	3.10
Zimbabwe	6,360	2.02
Overall	314,099	100

Table 2: Descriptive characteristics of women aged 15-49 in sub-Saharan African countries

**Variables**	**Weighted frequency**	**Percentage (%)**
**Residence**		
Rural	213,371	67.93
Urban	100,728	32.07
**Maternal age (in years)**		
15-24	91,416	29.10
25-34	149,661	47.65
≥35	73,021	23.25
**Maternal education**		
No education	110,470	35.17
Primary	109,700	34.93
Secondary	79,648	25.36

Higher	14,281	4.55
**Household wealth status**		
Poorest	71,982	22.92
Poorer	67,690	21.55
Middle	62,682	19.96
Richer	59,357	18.90
Richest	52,288	16.68
**Media exposure**		
No	121,460	38.67
Yes	192,639	61.33
**Parity**		
One birth	49,888	15.88
Two to four births	163,314	51.99
Five births and above	100,896	32.12
**Perceived distance to health facility**		
A big problem	144,906	46.13
Not a big problem	169,193	53.87
**Preceding birth interval**		
Primiparous	75,343	23.99
< 2 years	44,353	14.12
2 – 4 years	156,572	49.85
≥ 5 years	37,831	12.04
**Maternal working status**		

No	113,721	36.21
Yes	200,378	63.79
**Place of delivery**		
Home	118,609	37.76
Health facility	195,490	62.24
**ANC**		
No	123,981	39.47
Yes	190,118	60.53

Table 3: Multilevel binary logistic regression analysis of factors associated with perinatal mortality in SSA

**Variables**	**Null model**	**Model II (AOR with 95% CI)**	**Model III (AOR with 95% CI)**	**Model IV (AOR with 95% CI)**
**Maternal age (in years)**				
15-24		1		1
25-34		0.97 (0.92, 1.02)		0.96 (0.91, 1.01)
≥ 35		1.15 (1.07, 1.23)		1.13 (1.06, 1.21) *
**Maternal education status**				
No education		1		1
Primary		0.95 (0.91, 1.00)		1.05 (0.99, 1.10)
Secondary		0.89 (0.84, 0.95)		0.94 (0.89, 1.00)
Higher		0.79 (0.70, 0.89)		0.82 (0.73, 0.93) **

**Household wealth status**				
Poorest		1		1
Poorer		0.95 (0.90, 1.01)		0.94 (0.90, 1.00)
Middle		0.93 (0.88, 0.98)		0.93 (0.88, 0.98) **
Richer		0.91 (0.86, 0.97)		0.93 (0.87, 0.99) **
Richest		0.88 (0.82, 0.94)		0.92 (0.85, 1.00)
**Media exposure**				
No		1.16 (1.11, 1.21)		1.12 (1.08, 1.17) *
Yes		1		1
**Parity**				
< 2		1		1
2-4		1.34 (1.24, 1.45)		1.35 (1.25, 1.47) *
≥5		1.64 (1.49, 1.81)		1.69 (1.53, 1.86) **
**Preceding birth interval**				
< 2 years		1		1
2-4 years		0.71 (0.68, 0.75)		0.70 (0.67, 0.74) **
≥ 5 years		0.91 (0.85, 0.97)		0.91 (0.84, 0.97) *
Primiparous		1.20 (1.12, 1.30)		1.20 (1.12, 1.30)
**Place of delivery**				
Health facility delivery		1		1
Home delivery		1.10 (1.05, 1.15)		1.10 (1.06, 1.15) *
**ANC visit**				
No		1		1

Yes		0.66 (0.63, 0.69)		0.66 (0.63, 0.69) **
**Residence**				
Urban			1	1
Rural			1.14 (1.09, 1.19)	1.08 (1.02, 1.13) *
**SSA region**				
East Africa			1	1
West Africa			1.32 (1.27, 1.38)	1.30 (1.24, 1.36) **
Southern Africa			1.02 (0.89, 1.17)	1.11 (0.96, 1.27)
Central Africa			1.10 (1.05, 1.16)	1.05 (1.00, 1.11)
**Distance to health facility**				
Not big problem			1	1
Big problem			1.03 (0.99, 1.07)	1.06 (1.02, 1.10) *
**Random effect analysis results**				
LLR	-51098.94	-50466.46	-50998.78	-50390.07
Deviance	102197.9	100932.92	101997.6	100780.1

**ANC: Antenatal Care, AOR: Adjusted Odds Ratio, CI: Confidence Interval, LLR: Log-likelihood Ratio, * $p < 0.05$, ** $p < 0.01$*

Figure legend

Figure 1: Perinatal mortality rate across countries in SSA

Author response

PLOS ONE Journal

Manuscript title: Factors associated with perinatal mortality in Sub-Saharan Africa: A multilevel analysis

Manuscript ID: PONE-D-24-24034

Dear editor/reviewer.

Dear all,

We would like to thank you for the constructive, building, and improvable comments on this manuscript that would improve the content of the manuscript. We considered each comment and clarification question of editors and reviewers on the manuscript thoroughly. Our point-by-point responses for each comment and question are described in detail on the following pages. Further, the details of changes were shown by track changes in the supplementary document attached.

Reviewer comments

1. Abstract

These variables will have to be put in special case, e.g., Media Exposure, or with inverted commas, e.g., "Media Exposure" (as it was in the report), so that it is clear to the reader...

Authors' response: Thank you reviewer for the comments. We have accepted all the comments and revised them according to your suggestions. (See the Revised manuscript)

2. Background

- It would be good to put some figures here, so the reader can focus on differences between regions.

Authors' response: Thank you reviewer for the suggestions. We have added the figures for the perinatal mortality rate. "Annually, nearly 7 million perinatal deaths occur globally, including 3 to 4 million stillbirths and 3 million early neonatal deaths, with approximately 99% of these deaths taking place in low- and middle-income regions, predominantly in sub-Saharan Africa". (See the revised manuscript)

- By which institution has this been advocated...WHO?...Important to instruct the reader...

Authors' response: Thank you reviewer for the comment. We have included which institution advocated the ENAP. "Several interventions to lower the Perinatal Mortality Rate (PMR) have

been implemented globally, aiming to reduce stillbirths and neonatal deaths to 12 per 1,000 births by 2030 through the adoption of the Every Newborn Action Plan (ENAP) [12, 26, 27]. Launched in 2014 by the World Health Organization (WHO) and the United Nations Children's Fund (UNICEF), ENAP seeks to prevent newborn deaths and stillbirths while improving maternal and child health worldwide. However, PMR in sub-Saharan Africa remains alarmingly high compared to developed nations". (See the Revised manuscript).

3. Methods

- The sentence is unclear. Here it seems to mean that the birth record dataset was also used to extract data on "independent variables". Needs rephrasing.

Authors' response: Thank you reviewer for the comments. We used the Births Record (BR) dataset. As you know there are several DHS datasets including the women, men, kids, births, couples, and household datasets, and the type of dataset to be used depends on the objective of the research. For the child and birth outcomes, we can use either the births record or kids record data. As per the DHS recode manual and guide to statistics, we used the BR dataset to extract the outcome and independent variables. "Data on perinatal mortality and independent variables were extracted from the Births Record (BR) dataset.".

- Again, variables should be written differently from the sentence words, e.g., by capitalizing them, e.g., No, Yes, or better, by using inverted commas (e.g., "Yes", "No").

Authors' response: Thank you reviewer for the comment. We have accepted the comment and made the revision. (See the Revised manuscript)

- "and deviance"? Is this an incomplete sentence? Please re-word/improve.

Authors' response: Thank you for the comment. We have revised it. We used the deviance (-2LLR) for model comparison because the models were nested models where the lower the deviance value the better the model fits the data. A model with the lowest deviance value was chosen as the best-fitted model. We have added some details about deviance in the methods. "Four models were fitted, and model comparison was conducted using deviance (-2 Log-likelihood Ratio (-2LLR)), with the lowest value indicating the best-fitting model for the data. Deviance, expressed as the -2 Log-likelihood Ratio (-2LLR), is a statistical metric used to compare nested models in logistic regression and other likelihood-based approaches [31]. For nested models, it assesses whether the more complex model provides a better fit to the data than a simpler model by determining if the additional parameters in the complex model significantly enhance its

performance. When it approaches zero, it indicates that the model fits the data exceptionally well". (See the Revised manuscript)

- De-identified DHS data, with what?

Author's response: Thank you for the comment. We have revised the ethical consideration statement. In DHS, data was de-identified using data anonymization and removal of personal identifiers to ensure the participant's privacy and confidentiality. "For this study, we have received an authorization letter from the Measure DHS program for using the data. DHS provides publicly available de-identified data, so ethical approval is not needed. DHS employs several approaches to de-identify the data to ensure the privacy and confidentiality of respondents, such as data anonymization and the removal of personal identifiers". (See the Revised manuscript)

4. **Results**

   1. For the benefit of the reader, develop/explain this more, e.g., a way to generalize the residual sums of squares, to show the "goodness of fit"...etc.

   Author's response: Thank you for raising your concern. For our study, we used LLR and deviance for model comparison. As you may know, deviance is -2 times the log-likelihood ratio of the model. Given our outcome variable is binary /categorical, the model fitness/adequacy is ideally assessed using LLR because the model is based on the maximum likelihood estimation, unlike the linear regression where least square regression is used. So, the residual sum of squares is the key metric for assessing the model fitness of linear regression models where the residuals are the difference between the predicted and observed values. Therefore, the residual sum of squares can not be used for the logistic regression model. So, for our study, we used the model fitness using the LR test and deviance. For further reading see https://stats.stackexchange.com/questions/237702/comparing-models-using-the-deviance-and-log-likelihood-ratio-tests.

General comments

We have accepted all the editorial comments and revised them accordingly. (See the revised manuscript)
