# Supplementary material for: Factors associated with perinatal mortality in sub-Saharan Africa: A multilevel analysis
Source: PLoS One. 2024 Nov 21;19(11):e0314096. doi: 10.1371/journal.pone.0314096 (PMC11581209; doi:10.1371/journal.pone.0314096)
Supplement: Attachment — Submitted filename: Author response.docx [file pone.0314096.s002.docx]

**Author response**

**PLOS ONE Journal**

# **Manuscript title**: Factors associated with perinatal mortality in Sub-Saharan Africa: A multilevel analysis

**Manuscript ID:**  PONE-D-24-24034

**Dear editor/reviewer.**

Dear all,

We would like to thank you for the constructive, building, and improvable comments on this manuscript that would improve the content of the manuscript. We considered each comment and clarification question of editors and reviewers on the manuscript thoroughly. Our point-by-point responses for each comment and question are described in detail on the following pages. Further, the details of changes were shown by track changes in the supplementary document attached.

**Reviewer comments**

1. Abstract

These variables will have to be put in special case, e.g., Media Exposure, or with inverted commas, e.g., "Media Exposure" (as it was in the report), so that it is clear to the reader...

**Authors’ response**: Thank you reviewer for the comments. We have accepted all the comments and revised them according to your suggestions. (See the Revised manuscript)

2. Background

- It would be good to put some figures here, so the reader can focus on differences between regions.

**Authors’ response**: Thank you reviewer for the suggestions. We have added the figures for the perinatal mortality rate. "Annually, nearly 7 million perinatal deaths occur globally, including 3 to 4 million stillbirths and 3 million early neonatal deaths, with approximately 99% of these deaths taking place in low- and middle-income regions, predominantly in sub-Saharan Africa". (See the revised manuscript)

- By which institution has this been advocated...WHO?...Important to instruct the reader...

**Authors’ response**: Thank you reviewer for the comment. We have included which institution advocated the ENAP. “Several interventions to lower the Perinatal Mortality Rate (PMR) have been implemented globally, aiming to reduce stillbirths and neonatal deaths to 12 per 1,000 births by 2030 through the adoption of the Every Newborn Action Plan (ENAP) [12, 26, 27]. Launched in 2014 by the World Health Organization (WHO) and the United Nations Children's Fund (UNICEF), ENAP seeks to prevent newborn deaths and stillbirths while improving maternal and child health worldwide. However, PMR in sub-Saharan Africa remains alarmingly high compared to developed nations”. (See the Revised manuscript).

3. Methods

- The sentence is unclear. Here it seems to mean that the birth record dataset was also used to extract data on "independent variables". Needs rephrasing.

**Authors’ response**: Thank you reviewer for the comments. We used the Births Record (BR) dataset. As you know there are several DHS datasets including the women, men, kids, births, couples, and household datasets, and the type of dataset to be used depends on the objective of the research. For the child and birth outcomes, we can use either the births record or kids record data. As per the DHS recode manual and guide to statistics, we used the BR dataset to extract the outcome and independent variables. "Data on perinatal mortality and independent variables were extracted from the Births Record (BR) dataset.”.

- Again, variables should be written differently from the sentence words, e.g., by capitalizing them, e.g., No, Yes, or better, by using inverted commas (e.g., "Yes", "No").

**Authors’ response**: Thank you reviewer for the comment. We have accepted the comment and made the revision. (See the Revised manuscript)

- "and deviance"? Is this an incomplete sentence? Please re-word/improve.

**Authors’ response**: Thank you for the comment. We have revised it. We used the deviance (-2LLR) for model comparison because the models were nested models where the lower the deviance value the better the model fits the data. A model with the lowest deviance value was chosen as the best-fitted model. We have added some details about deviance in the methods. "Four models were fitted, and model comparison was conducted using deviance (-2 Log-likelihood Ratio (-2LLR)), with the lowest value indicating the best-fitting model for the data. Deviance, expressed as the -2 Log-likelihood Ratio (-2LLR), is a statistical metric used to compare nested models in logistic regression and other likelihood-based approaches [31]. For nested models, it assesses whether the more complex model provides a better fit to the data than a simpler model by determining if the additional parameters in the complex model significantly enhance its performance. When it approaches zero, it indicates that the model fits the data exceptionally well”. (See the Revised manuscript)

- De-identified DHS data, with what?

**Author's response**: Thank you for the comment. We have revised the ethical consideration statement. In DHS, data was de-identified using data anonymization and removal of personal identifiers to ensure the participant's privacy and confidentiality. "For this study, we have received an authorization letter from the Measure DHS program for using the data. DHS provides publicly available de-identified data, so ethical approval is not needed. DHS employs several approaches to de-identify the data to ensure the privacy and confidentiality of respondents, such as data anonymization and the removal of personal identifiers”. (See the Revised manuscript)

4. **Results**
1. For the benefit of the reader, develop/explain this more, e.g., a way to generalize the residual sums of squares, to show the "goodness of fit"...etc.

**Author's response:** Thank you for raising your concern. For our study, we used LLR and deviance for model comparison. As you may know, deviance is -2 times the log-likelihood ratio of the model. Given our outcome variable is binary /categorical, the model fitness/adequacy is ideally assessed using LLR because the model is based on the maximum likelihood estimation, unlike the linear regression where least square regression is used. So, the residual sum of squares is the key metric for assessing the model fitness of linear regression models where the residuals are the difference between the predicted and observed values. Therefore, the residual sum of squares can not be used for the logistic regression model. So, for our study, we used the model fitness using the LR test and deviance. For further reading see <https://stats.stackexchange.com/questions/237702/comparing-models-using-the-deviance-and-log-likelihood-ratio-tests>.

General comments

We have accepted all the editorial comments and revised them accordingly. (See the revised manuscript)
